# Comprehensive aptamer-based screening identifies a spectrum of urinary biomarkers of lupus nephritis across ethnicities

**DOI:** 10.1038/s41467-020-15986-3

**Published:** 2020-05-04

**Authors:** Samantha Stanley, Kamala Vanarsa, Samar Soliman, Deena Habazi, Claudia Pedroza, Gabriel Gidley, Ting Zhang, Shree Mohan, Evan Der, Hemant Suryawanshi, Thomas Tuschl, Jill Buyon, Chaim Putterman, Chi Chiu Mok, Michelle Petri, Ramesh Saxena, Chandra Mohan

**Affiliations:** 1 Department Biomedical Engineering, University of Houston, Houston, TX USA; 2 Rheumatology and Rehabilitation Department, Faculty of Medicine, Minia University, Minya, Egypt; 3 Center for Clinical Research and Evidence-Based Medicine, University of Texas Health Science Center at Houston, Houston, TX USA; 4 Department of Rheumatology, Albert Einstein College of Medicine, Bronx, NY USA; 5 Department of Molecular Biology, Rockefeller University, New York, NY USA; 6 Department of Rheumatology, New York University, New York, NY USA; 7 Department of Medicine, Tuen Mun Hospital, New Territories, Hong Kong, China; 8 Division of Rheumatology, Johns Hopkins University School of Medicine, Baltimore, MD USA; 9 University Hospital Kidney & Liver Clinic, University of Texas Southwestern Medical Center, Dallas, TX USA

**Keywords:** Proteomics, Diagnostic markers, Lupus nephritis, Population screening

## Abstract

Emerging urinary biomarkers continue to show promise in evaluating lupus nephritis (LN). Here, we screen urine from active LN patients for 1129 proteins using an aptamer-based platform, followed by ELISA validation in two independent cohorts comprised of 127 inactive lupus, 107 active LN, 67 active non-renal lupus patients and 74 healthy controls, of three different ethnicities. Urine proteins that best distinguish active LN from inactive disease are ALCAM, PF-4, properdin, and VCAM-1 among African-Americans, sE-selectin, VCAM-1, BFL-1 and Hemopexin among Caucasians, and ALCAM, VCAM-1, TFPI and PF-4 among Asians. Most of these correlate significantly with disease activity indices in the respective ethnic groups, and surpass conventional metrics in identifying active LN, with better sensitivity, and negative/positive predictive values. Several elevated urinary molecules are also expressed within the kidneys in LN, based on single-cell RNAseq analysis. Longitudinal studies are warranted to assess the utility of these biomarkers in tracking lupus nephritis.

## Abstract (web summary)

Developing noninvasive diagnostic biomarkers for lupus nephritis (LN) diagnosis is an important clinical goal. Here the authors identify urinary proteins correlated with active LN and disease severity, which differ across ethnicities but collectively outperform the current clinical method.

## Introduction

Lupus nephritis (LN), one of the most severe complications of systemic lupus erythematosus (SLE), is a condition where the kidneys become inflamed and eventually lose function. It is estimated that ~60% of all SLE patients will develop LN^1^, and in 10–15% of those patients the disease will progress to end-stage renal disease (ESRD)^1^. The current gold standard for diagnosis of renal involvement is a renal biopsy; while biopsies are highly informative, they cannot be serially repeated and come with attendant concerns, including the invasive nature of the procedure, and the possibility that the sample taken may not be representative of the entire kidney. It has been documented that early detection and prompt treatment can have a significant impact on morbidity and mortality in LN^2^, but current diagnostic techniques are not optimal for early detection. Hence, an easily measurable biomarker for LN with high predictive value is highly desirable, and this has sparked significant research interest in this direction.

SLE and LN are both heavily influenced by genetics^3^, and African-Americans are three times more likely to develop SLE than Caucasians^4^. Likewise, disease manifestations are variably expressed among patients, with African-Americans being more likely to develop ESRD^5^, although influence from environmental triggers or socioeconomic factors cannot be ruled out^5,6^. Although patient demographics are widely known to affect SLE disease manifestations and outcomes, there are virtually no studies investigating this phenomenon in the context of disease biomarkers; most SLE biomarkers studies focus on one demographic group or all ethnic groups combined, which yield results that may not be equally predictive in all demographic groups of SLE patients.

Traditional biomarker discovery study design is typically based on prior understanding of established pathophysiological pathways underlying LN, with a focus on selected molecules (such as specific growth factors, cytokines, and chemokines) directly related to those pathways, which stamps a bias on the types of biomarkers identified. In contrast, large-scale proteomic approaches have transformed the discovery of urinary biomarkers from a highly skewed search to a comprehensive unbiased screen. A couple of studies in LN have utilized non-targeted proteomic approaches, including isobaric tag for relative and absolute quantitation (iTRAQ) mass spectrometry^7^, and electrospray ionization quadrupole time-of-flight tandem mass spectrometry (ESI-Q-TOF MS/MS)^8^.

In contrast to the above mass spectrometry-based approaches, which typically uncover high abundance proteins, affinity-based approaches using various ligands (such as antibodies) have the potential to uncover lower abundance proteins, due to the use of specific, high-affinity ligands. A few screening studies in LN have utilized affinity-based techniques such as antibody-based or aptamer-based arrays, with only one study using antibody-based arrays in the context of SLE^9^. The aptamer-based screen used in the present study has the power of simultaneous interrogation of over 1100 unique proteins, with a dynamic ranger larger than that of a traditional enzyme-linked immunosorbent assay (ELISA). It is based on specially designed aptamers, which are synthetic, single-stranded DNA-based molecular recognition elements, to selectively recognize and quantify a wide spectrum of proteins in body fluids or cell lysates^10^. This platform has been successfully applied in biomarker screens of several diseases, including Alzheimer’s disease^11^, pulmonary tuberculosis^12^, and others^13–16^, but not in autoimmune diseases. In the current study, this aptamer-based screen is applied to identify potential urinary biomarkers in LN. Identified biomarker candidates are further validated in independent cross-sectional cohorts to validate the screening hits. Interestingly, the validated molecules exhibit striking ethnic-group-specific differences in their biomarker potential. Moreover, several of the urinary biomarkers elevated in LN urine are expressed within the kidneys in LN, either within resident renal cells or infiltrating immune cells.

## Results

### Aptamer-based screening of LN urine

**Fig. 1:**
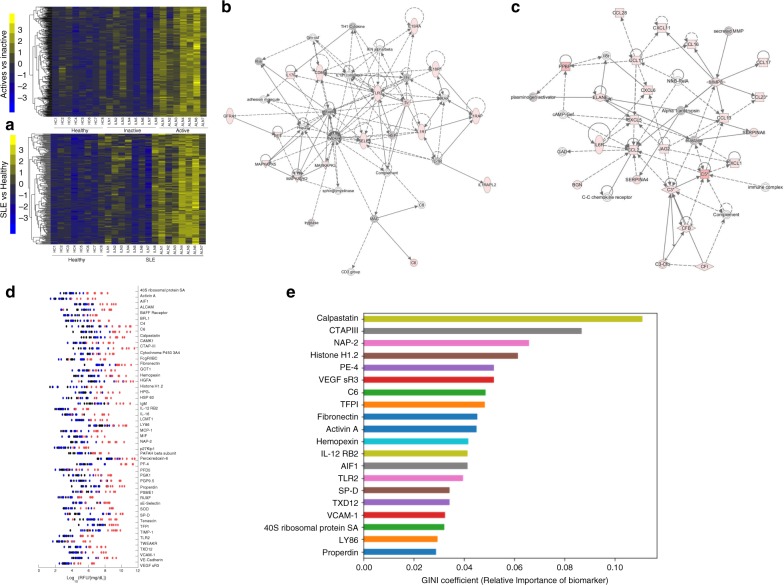
Aptamer-based screening of lupus nephritis urine samples for 1129 proteins. **a** Heatmap representation of aptamer-based screening results of the 1129 proteins analyzed in 23 human urine samples (7 active LN, 8 inactive SLE, 8 healthy controls). 284 urinary proteins were found to be elevated (*p* < 0.05, fold-change ≥2, Mann–Whitney *U*-test) when comparing active LN to inactive SLE (top), while 326 urinary proteins were elevated (*p* < 0.05, fold-change ≥2; Mann–Whitney *U*-test) when comparing all SLE subjects to healthy controls (bottom). Each map shows the relative concentrations of these proteins after normalizing against urinary creatinine. Each column represents a patient sample, while rows correspond to a creatinine-normalized protein level measured using the screening assay. Proteins that are above the mean value (for each biomarker) are yellow, while those below are blue, with proteins comparable to the mean value are black. **b**, **c** The proteins that were significantly elevated in the urine of patients with active LN clustered into 20 pathways with at least 10 upregulated proteins each, as determined by Ingenuity Pathway Analysis, of which 2 are displayed. Additional pathways are included in Supplementary Data. Molecules elevated in LN urine are shaded pink. Documented and putative interactions between the displayed molecules are indicated by solid and interrupted arrows, respectively, based on literature review. **d** The 50 urine proteins (ranked in order of fold-change) that were significantly elevated in active LN compared to inactive SLE are displayed. Further details regarding these proteins are in included in Supplementary Data (red dots represent active LN; blue dots represent inactive SLE; black dots represent healthy controls). **e** Random forest classification analysis identification of the 20 most discriminatory urine proteins with the largest impact on distinguishing the study groups, ordered by their GINI coefficient. Source data are provided as a Source Data file.

Urine samples from 23 human subjects (7 active LN, 8 inactive SLE, 8 healthy controls, all female, age range 23–42 years) were initially screened for the levels of 1129 distinct human proteins using a pre-fabricated aptamer-based-targeted proteomic assay, that is commercially available^10^. In this assay, streptavidin-coated beads labeled with 1129 unique aptamers are added to each urine sample to allow them to bind to their designated protein targets^17^. After incubation, the beads are removed from the sample, the proteins attached to the aptamers are biotinylated and all aptamer–protein complexes are cleaved from the initial streptavidin beads and re-coupled to a new bead, with the biotinylated protein attaching to the bead. The aptamers are then removed from the beads and quantitated using a DNA microarray^10^. The assay readouts (measured as relative fluorescence units or RFU) were normalized to urinary creatinine levels. In this assay, 326 proteins were significantly elevated in SLE urine compared to healthy control urine, while 284 proteins were significantly elevated in active LN urine compared to inactive SLE urine, with 198 proteins overlapping between these two comparisons, as displayed in the heatmap in Fig. 1a.

The proteins that were significantly elevated in the urine of patients with active LN clustered into 20 pathways with at least 10 upregulated proteins each, as determined by Ingenuity Pathway Analysis. One of these pathways was enriched for several molecules involved in inflammation, including IL1R1, IL1RAP, IL1RAPL2, IL15RA, IL17F, IL18R1, E-selectin, TLR2, CD86, and several signaling molecules, including MAKAPK2, MAPKAPK3, and MAPKAPK5, all of which were significantly elevated in the urine of patients with active LN, as indicated by the pink-colored nodes in Fig. 1b. Another network of urine proteins elevated in LN urine included various complement proteins (C3, C5, CFB, CFI), several chemokines (CCL2 (MCP-1), CCL11 (Eotaxin), CCL13, CCL16, CCL17 (TARC), CCL23 (MIP-3), CCL28, CXCL1 (KC; Gro1), CXCL4 (PF-4), CXCL5, CXCL6, CXCL11 (iTAC)), and other molecules implicated in inflammation, including IL6R, MMP8, and SERPINA4 (Kallistatin; alpha-1-anti-trypsin), as displayed in Fig. 1c. A third upregulated network interconnected several members of the TNF/TNF-receptor superfamily, including TNFSF12 (TWEAK), TNFRSF12A (TWEAK-R, Fn14), TNFSF4 (OX40L), TNFSF14 (LIGHT), TNFSF6 (FasLG; CD95L), TNFRSF6B, TNFRSF13c (BAFF-R), TNFRFSF9 (41BBL; CD137), TNFRSF21 (CD358; DR6), and TNFRSF25 (DR3), as shown in Supplementary Fig. 1a. Other upregulated pathways include proteins important for extracellular matrix turnover and/or fibrosis (Collagen, COL8A, TNNI2, PDGFB, PDGFAb, PDGFBB, CTGF), metalloprotease family members (TIMP1, TIMP3, ADAM9, ADAMTS1, ADAMTS4), NOTCH family members (NOTCH2, NPTCH3, DLL1), and cadherins (CDH2, CDH5, CDH15), as displayed in Supplementary Fig. 1b and 1c.

Next, to validate the observed elevations in the primary screen, we limited further analysis to the 50 proteins (ranked in order of fold-change) that were significantly elevated in active LN compared to inactive SLE, as displayed in Fig. 1d, and detailed in Supplementary Table 1. As can be seen in this table, most of these proteins were significantly elevated in the urine of active LN patients (compared to inactive disease), even after multiple testing correction (*q* < 0.05). Random forest analysis, a machine learning algorithm, implicated urine calpastatin, CTAP-III, NAP-2, Histone H1.2, PF-4, VEGF sR3, C6, and TFPI as some of the most discriminatory molecules with the largest impact on distinguishing the study groups (Fig. 1e). Of these top 50 proteins, 21 proteins were not pursued further for the following reasons, as detailed in Supplementary Table 2: average intensity levels of protein in all subject groups were <1000 RFU (IL-12Rb2, p27Kip1, PFD5, RUXF, TLR2, TWEAKR, VEGF sR3), previous studies had already documented the elevations of these markers (IgM, MIF), or there was a strong correlation (correlation coefficient *r* > 0.95) with another chosen protein (CAMK1, CTAP-III, Cytochrome P450, GOT1, HGFA, Histone H1.2, LCMT1, PAFAHβ1, PGP9.5, PSME1, SP-D, TXD-12). ELISA kits were purchased for the remaining 29 proteins. Of these, the purchased ELISA kits did not work for urine AIF1 and 40s ribosomal protein SA (Supplementary Fig. 2).

### Validation of proteomic hits in a primary independent cohort

With the remaining 27 biomarker candidates (for which functional ELISA kits were identified), pilot ELISA testing was carried out using a limited cohort of 36 subjects, comprises 12 active LN, 12 inactive SLE, and 12 healthy controls. With 15 of these 27 tested proteins, the urine levels in active LN were not significantly higher than that in inactive SLE (as listed in Supplementary Table 2). In contrast, 12 urinary proteins continued to show significantly higher levels in active LN in this pilot ELISA test (as indicated in bold in Supplementary Table 2), and were hence pursued further. These proteins were also tested in a subset of urine samples included in the aptamer-based screening assay in order to assess the correlation between the two platforms—ELISA readouts and the aptamer-based screening results; these data are outlined in Table 1. For most proteins, the correlation of the biomarker results between the aptamer screening result and the ELISA-validation assay exceeded 0.8, with these values exceeding 0.9 for ALCAM, peroxiredoxin-6, PF-4, properdin, and VCAM-1 (Table 1).

**Table 1:** Correlation between aptamer screen and ELISA assay results.

Molecule	Pearson correlation
ALCAM	0.926***
BFL-1	N/A
Calpastatin	0.728*
FcgRIIB/C	N/A
Hemopexin	0.752*
MCP-1	0.771*
Peroxiredoxin-6	0.969***
PF-4	0.977***
Properdin	0.927***
sE-Selectin	N/A
TFPI	0.805*
VCAM-1	0.981***

Twenty-four urine samples (8 active LN, 8 inactive SLE, 8 healthy controls) were assayed on both platforms (aptamer-based screening and ELISA assays). Listed are the Spearman correlation coefficients between the two platforms, for each protein, and the associated significance (*p < 0.05; ***p < 0.001).

N/A Not done.

**Fig. 2:**
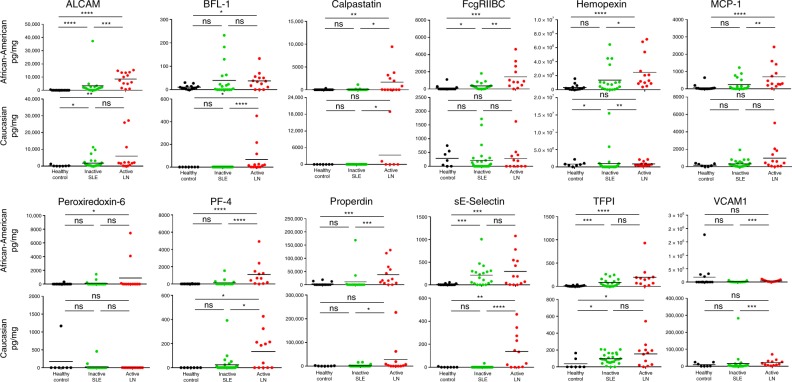
ELISA validation of elevated urinary proteins in African-American and Caucasian lupus nephritis patients. Plotted are ELISA-validation results for 12 urine proteins in African-American and Caucasian subjects. Each protein was tested using the following sample group: 27 active SLE samples (14 African-American, 13 Caucasian), 47 inactive SLE samples (19 African-American, 28 Caucasian), and 21 healthy controls (14 African-American, 7 Caucasian). The plots show the mean concentration in urine for each disease group after normalizing against urinary creatinine. *p < 0.05, **p < 0.01, and ***p < 0.001, as determined using Mann–Whitney *U*-test. Further details regarding these proteins are included in Supplementary Data. Source data are provided as a Source Data file.

The 12 shortlisted urine proteins from the 1129 initially screened were ELISA tested in a cross-sectional cohort of 95 subjects with African-American and Caucasian ethnicity (comprised of 47 inactive SLE patients, 27 active LN patients, and 21 healthy controls), and creatinine-normalized (Fig. 2). The demographic attributes, clinical features, and medication history of these subjects are presented in Table 2, parsed by ethnic group. Each urinary protein was analyzed to determine how well it distinguished SLE patients from healthy controls, and active LN from inactive SLE, using receiver operating curve analysis. 10 of the 12 urine proteins interrogated outperformed traditional laboratory measures including C3/C4 and anti-dsDNA in discriminating active LN from inactive SLE, with improved AUC values and statistical significance, as detailed in Table 3. Among these, urine VCAM-1, PF-4, and properdin performed the best in distinguishing active from inactive disease, with AUC values ≥79%. These three urine proteins exhibited fold-increase values ranging from 2.4 to 8.9, comparing patients with active LN to those with inactive disease. They also exhibited superior sensitivity, NPV and PPV values compared to the traditional yardsticks, and several of the other proteins interrogated. As detailed in Table 4, urine ALCAM, BFL-1, calpastatin, hemopexin, MCP-1, PF-4, properdin, sE-selectin, and VCAM-1 maintained significant association with active renal disease status, after adjusting for race, age, prednisone usage, and multiple testing correction, as determined by multivariable logistic regression analysis.

**Table 2:** Patient cohort used for the cross-sectional validation studies.

	**African-American cohort**			**Caucasian cohort**		
	**Active LN**	**Inactive SLE**	**Healthy control**	**Active LN**	**Inactive SLE**	**Healthy control**
	*N* = 14	*N* = 19	*N* = 14	*N* = 13	*N* = 28	*N* = 7
Age (years)	31.2 ± 8.6	33.1 ± 10.3	32.2 ± 5.1	44.8 ± 10.8	48.9 ± 10.7	50.1 ± 5.7
Female, no. (%)	14 (100%)	19 (100%)	14(100%)	13 (100%)	28(100%)	7(100%)
SLEDAI, median (IQR)	11(10–14)	2 (0–4)	N/A	12(10–12)	0 (0–0)	N/A
rSLEDAI, median (IQR)	8 (8–12)	0 (0–0)	N/A	8 (8–8)	0 (0–0)	N/A
PGA, median (IQR)	2.1 (1.9–2.5)	1 (0.5–1.2)	N/A	1.5 (1.5–1.8)	0.5 (0–0.6)	N/A
Protein:Cr ratio (mg/mg)	2.7 ± 1.5	0.4 ± 0.3	N/A	1.3 ± 1.0	0.2 ± 0.1	N/A
eGFR (ml/min/1.73 m2)	121 ± 40	100 ± 45	N/A	83 ± 24	77 ± 26	N/A
Serum creatinine (mg/dl)	0.8 ± 0.3	1 ± 0.5	N/A	0.9 ± 0.2	0.9 ± 0.3	N/A
Anti-dsDNA+ve/total tested	7/14	6/17	N/A	7/13	1/28	N/A
Hypocomplementemia/total	5/13	5/19	N/A	7/13	4/28	N/A

Concurrent medication use, *n* (%)						
Prednisone	10 (71%)	15 (79%)	N/A	10 (77%)	9 (32%)	N/A
Immunosuppressants	12 (86%)	12 (63%)	N/A	7 (54%)	16 (57%)	N/A
Plaquenil	14 (100%)	18 (95%)	N/A	8 (62%)	23 (82%)	N/A
NSAID	3 (21%)	2 (11%)	N/A	3 (23%)	1 (4%)	N/A
Anti-hypertensives	10 (71%)	12 (63%)	N/A	12 (92%)	23 (82%)	N/A
Diuretic	4 (29%)	5 (26%)	N/A	3 (23%)	4 (14%)	N/A
ACE inhibitor or ARB	8 (57%)	11 (58%)	N/A	8 (62%)	18 (64%)	N/A
Statin	3 (21%)	3 (16%)	N/A	3 (23%)	16 (57%)	N/A

Anti-dsDNA+ve refers to number of subjects who were positive for anti-dsDNA antibodies.

Source data are provided as a Source Data file.

**Table 3:** Urine Cr-normalized biomarker levels in a primary validation cohort.

**Urine protein**	Fold-change^a^							
	Active/inactive	SLE/healthy	**Comparison of active LN vs inactive SLE**					
			**Cut-off**	**ROC AUC**	**Sensitivity**	**Specificity**	**NPV**	**PPV**
ALCAM	3.0***	40.7****	4875	0.74***	0.52	0.92	0.78	0.77
BFL-1	3.3***	4.3	11	0.74****	0.70	0.81	0.68	0.83
Calpastatin	63**	51.0*	325	0.74****	0.52	0.98	0.93	0.78
FcγRIIBC	3.2*	2.7	367	0.69**	0.59	0.79	0.62	0.77
Hemopexin	1.5***	2.8	327459	0.76****	0.96	0.57	0.57	0.96
MCP-1	2.9**	4.4**	203	0.71***	0.78	0.62	0.54	0.83
Peroxiredoxin-6	7.2	2.8	1	0.58	0.30	0.87	0.57	0.68
PF-4	8.9****	84.7**	39	0.81****	0.78	0.83	0.72	0.87
Properdin	6.2**	6.2	2062	0.79****	0.74	0.89	0.80	0.86
sE-Selectin	2.6**	22.2*	3	0.73****	0.82	0.66	0.58	0.86
TFPI	1.9	6.3****	146	0.65*	0.63	0.75	0.59	0.78
VCAM-1	2.5****	0.2	10324	0.85****	0.82	0.79	0.69	0.88
C3	0.9	N/A	146	0.39	0.19	0.92	0.56	0.66
C4	1	N/A	30	0.49	0.33	0.81	0.50	0.68
Anti-DNA	3.7	N/A	40	0.66**	0.52	0.81	0.61	0.75

The primary validation cohort of 95 subjects comprise 47 inactive SLE patients,

27 active LN patients, and 21 healthy controls.

Source data are provided as a Source Data file.

^a^*p*-values by Mann–Whitney *U*-test (*p < 0.05; **p < 0.01; ***p < 0.001; ****p < 0.0001).

**Table 4:** Association of urine biomarkers with active LN status after adjusting for confounding factors.

	Active LN vs inactive SLE					
Protein	Race as co-factor		Race + age co-factors		Race + prednisone co-factors	
	*p*-value	*q*-value	*p*-value	*q*-value	*p*-value	*q*-value
ALCAM	*	*	*	*	*	ns
BFL-1	***	**	***	**	**	**
Calpastatin	***	***	***	***	***	**
FcgRIIBC	ns	ns	ns	ns	ns	ns
Hemopexin	**	**	**	**	**	**
MCP-1	**	*	**	*	**	*
Peroxiredoxin-6	ns	ns	ns	ns	ns	ns
PF-4	***	***	***	***	***	***
Properdin	***	***	***	***	***	***
sE-Selectin	**	**	**	**	**	**
TFPI	ns	ns	ns	ns	ns	ns
VCAM-1	**	**	**	**	**	**

***p < 0.001, **p < 0.01, *p < 0.05, ns = *p* > 0.05. The same nomenclature is used for significance after multiple testing correction (*q*-values).

**Fig. 3:**
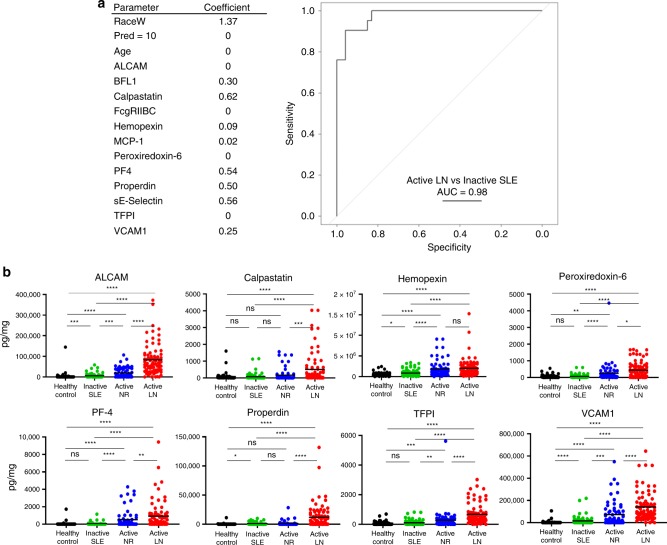
Testing the utility of multi-marker panels, and validity of markers in other ethnicities. **a** Lasso Regression Analysis to identify multi-marker panels. We performed Lasso regression analysis to identify multi-marker panels that may better predict active LN status in the primary validation cohort, as detailed in Methods. Besides the 12 urine proteins, we included race, age, and prednisone usage as additional variables. The combination with the best predictive model encompassed 8 of the 12 urine proteins, together with race, but excluded age and prednisone usage. This octuplex panel exhibited outstanding ability to discriminate active LN from inactive SLE with a ROC AUC value of 0.98. **b** Plotted are ELISA-validation results in a second cohort, comprised of Asian subjects. Each protein was tested using the following sample group: 80 active SLE samples, 80 inactive SLE samples, 67 active non-renal lupus samples, and 53 healthy controls. The plots show the mean concentration in urine for each disease group after normalizing against urinary creatinine. *p < 0.05, **p < 0.01, and ***p < 0.001, as determined using Mann–Whitney *U*-test. Source data are provided as a Source Data file.

Next, we performed Lasso regression analysis to identify multi-marker panels that may better predict active LN status in the primary validation cohort. Besides the 12 urine proteins, we included race, age, and prednisone usage as additional variables. The combination with the best predictive model encompassed 8 of the 12 urine proteins, together with race, but excluded age and prednisone usage. This octuplex panel exhibited outstanding ability to discriminate active LN from inactive SLE with a ROC AUC value of 0.98, as shown in Fig. 3a, and also identified race as a significant confounding variable.

### The diagnostic utility of biomarkers vary with ethnicity

As the above analyses identified race as a significant confounding factor, we examined the performance of these markers within each ethnic group. Among African-American patients, the best biomarkers that distinguished active LN from inactive disease, with statistical significance, were urine PF-4 (AUC = 0.88), VCAM-1 (AUC = 0.87), properdin (AUC = 0.85), ALCAM (AUC = 0.84), and FcgRIIBC (AUC = 0.82), followed by MCP-1, hemopexin, calpastatin and TFPI, all exceeding the performance of C3/C4 and anti-DNA (Supplementary Table 3 and Fig. 2). These same molecules also surpassed conventional laboratory yardsticks, in terms of assay sensitivity, PPV and NPV. Among the five urine proteins with the highest discriminatory potential (highest AUC values) in African-American patients, urine VCAM-1, and PF-4 exhibited the highest sensitivity (0.93) and PPV (0.93–0.94), whereas urine ALCAM and properdin exhibited the highest specificity (0.90–0.95) and NPV (0.86–0.92) (Supplementary Table 3).

Different biomarkers emerged as being useful among Caucasian patients, where the most discriminatory urine proteins were sE-selectin (AUC = 0.87), VCAM-1 (AUC = 0.84), BFL-1 (AUC = 0.81), and hemopexin (AUC = 0.80), followed by calpastatin, PF-4, and properdin (Supplementary Table 3 and Fig. 2). In particular, urine sE-selectin outperformed anti-DNA in terms of sensitivity, NPV and PPV, and matched anti-DNA in terms of specificity, when comparing Caucasian patients with active LN to those with inactive SLE (Supplementary Table 3). Also striking was the finding that the absolute urine levels of ALCAM and FcgRIIBC were significantly higher among African-American LN patients compared to Caucasian LN patients (*p* < 0.05, Mann–Whitney *U*-test; Fig. 2; Supplementary Table 3).

Given the observed variation in urine biomarkers across ethnicities, we next assayed these biomarkers in a second validation cohort of Asian patients. Power calculations based on ELISA results from the primary validation cohort indicate that we would need an average sample size of 52 per group (Supplementary Table 4). Hence, our secondary validation cohort was composed of 80 inactive SLE patients, 80 active LN patients, 67 active non-renal lupus patients, and 53 healthy controls. The demographic attributes, clinical features, and medication history of these subjects are presented in Supplementary Table 5. In this second validation cohort, four of the eight urine proteins interrogated, including ALCAM (AUC = 0.93), VCAM-1 (AUC = 0.92), TFPI (AUC = 0.88), and PF-4 (AUC = 0.83), outperformed C3/C4 and anti-dsDNA in discriminating active LN from inactive SLE, with improved AUC values and statistical significance, as detailed in Table 5 and Fig. 3b. The sensitivities and specificities of these biomarkers in the Asian cohort were also re-calculated using the same cut-off values used for the primary validation cohort, and this is presented in Supplementary Table 6. Even though the remaining four urine proteins, calpastatin, hemopexin, peroxiredoxin-6, and properdin, did not outperform anti-dsDNA in distinguishing active LN from inactive SLE patients, they were all significantly elevated in active LN when compared with inactive SLE patients, thus offering independent validation of the urinary biomarkers uncovered in this study, across multiple ethnic groups. More importantly, as plotted in Fig. 3b, several of the interrogated urine proteins were significantly elevated in active LN compared to active non-renal lupus in this cohort, alluding to their renal specificity. In particular, urine ALCAM (AUC = 0.84), calpastatin (AUC = 0.68), properdin (AUC = 0.75), TFPI (AUC = 0.78), and VCAM-1 (AUC = 0.73) exhibited the best ability to distinguish active renal involvement from active non-renal lupus. Interestingly, in patients with active LN, the urine levels of ALCAM, TFPI, and VCAM-1 were significantly higher in Asian patients when compared to either African-American or Caucasian patients (*p* < 0.05, Mann–Whitney *U*-test), alluding to potential genetic contributions underpinning these differences.

**Table 5:** Urine Cr-normalized biomarkers in the second (Chinese) validation cohort.

**Urine protein**	Fold-change^a^								
	Active/inactive	AR/ANR	SLE/healthy	**Comparison of active LN vs inactive SLE**^**a**^					
				**Cut-off**	**ROC AUC**	**Sens**.	**Spec**.	**NPV**	**PPV**
ALCAM	14****	4.3****	10.8****	15K	0.93****	0.9	0.91	0.9	0.91
Calpastatin	7.1****	3.1****	3.3	0	0.74****	0.66	0.78	0.7	0.75
Hemopexin	2.3****	1.1	2.6****	630K	0.74****	0.85	0.56	0.79	0.66
Peroxiredoxin-6	6.6****	1.8*	3.4****	0	0.75****	0.56	0.91	0.68	0.87
PF-4	24****	1.8**	11****	0	0.83****	0.74	0.88	0.77	0.86
Properdin	13****	10.8****	26	0	0.74****	0.61	0.84	0.68	0.79
TFPI	6.2****	2.4****	4.9****	200	0.88****	0.8	0.89	0.82	0.89
VCAM-1	8.9****	2.0****	21****	30,200	0.92****	0.9	0.88	0.9	0.88
C3	0.5****	0.8***	N/A	13	0.12	1	0	0	0.5
C4	0.5****	0.8**	N/A	3	0.19	1	0	0	0.5
Anti-DNA	2.4****	1.3**	N/A	215	0.82****	0.63	0.99	0.98	0.72

The cohort comprises 80 inactive SLE patients, 67 active non-renal (ANR),

80 active LN patients, and 53 healthy controls.

Source data are provided as a Source Data file.

^a^*p*-values by Mann–Whitney *U*-test (*p < 0.05; **p < 0.01; ***p < 0.001; ****p < 0.0001).

### Correlation with clinical and laboratory metrics

**Fig. 4:**
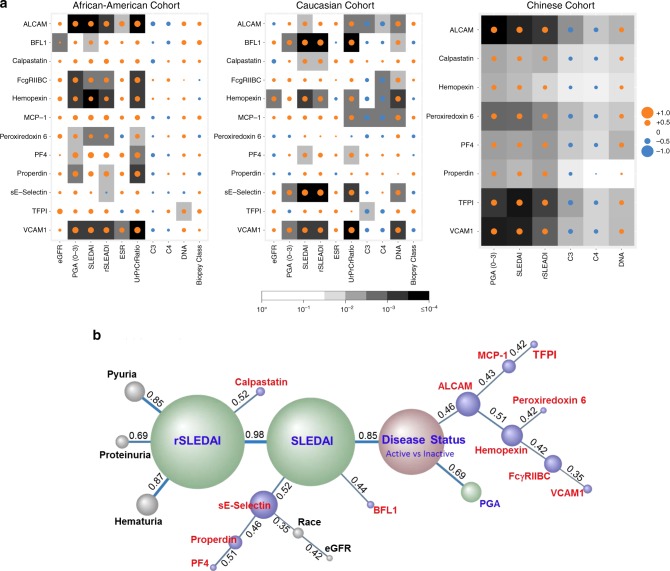
Correlation and association of urine biomarkers of lupus nephritis with clinical and laboratory indices. **a** Creatinine-normalized urine levels of the 12 proteins (listed vertically) were Pearson correlated with various clinical and laboratory yardsticks, as listed on the *x* axis, in both the African-American (14 active LN, 19 inactive SLE, 14 healthy controls), Caucasian (13 active LN, 28 inactive SLE, 7 healthy controls), and Asian (80 active LN, 80 inactive SLE, 67 active non-renal lupus, 53 healthy control) patient cohorts. It should be noted that the renal biopsy data included is not from concurrent biopsies, but from previous biopsies, executed 1-mo to 20 years before urine procurement. Positive and negative correlations are denoted by orange and blue circles respectively, while statistical significance is denoted using gray-scale boxes. **b** The levels of the 12 urine proteins in the combined cohort (27 active LN, 47 inactive SLE, and 21 healthy controls) and their respective clinical features were subjected to Bayesian network analysis using BayesiaLab. The network shown was constructed in an unsupervised manner, using the EQ algorithm and a structural coefficient of 0.4. The circular nodes that make up the Bayesian Network represent the variables of interest, including urine biomarkers (purple-colored), clinical indices (green-colored), other features (colored gray) and disease status (active LN vs inactive SLE vs no disease; colored brown). The size of each node denotes the “node force”, which is related to its impact on other nodes in the network, based on conditional probabilities. The links (arcs) that interconnect the nodes represent informational or causal dependencies among the variables, including the correlation coefficients between neighboring nodes (as indicated), with the thickness of the link being proportional to the correlation coefficient.

Most of the urine proteins that exhibited significant potential to discriminate active LN from inactive disease were also associated with disease activity indices such as SLEDAI, renal-SLEDAI (rSLEDAI), and physician global assessment (PGA). Nine of the validated urine proteins were significantly associated with rSLEDAI and PGA, after adjusting for race, age, prednisone usage, and multiple testing correction, as determined by multivariable linear regression analysis (Supplementary Table 7). This was further confirmed by a correlation analysis within each ethnic group of patients. Thus, within the African-American cohort, ALCAM, FcgRIIBC, hemopexin, peroxiredoxin-6, properdin, and VCAM-1 were positively correlated with PGA, SLEDAI, rSLEDAI scores, and proteinuria (*p* < 0.05, Mann–Whitney *U*-test; *R* > 0.4), with PF-4 showing a similar trend (as dot-plotted in Fig. 4a). Interestingly, urine ALCAM and VCAM-1 also positively correlated with ESR. These proteins exhibited poor correlation with C3/C4 and anti-DNA (Fig. 4a). Conversely, within the Caucasian cohort, urine BFL-1, sE-Selectin, and VCAM- 1 displayed a positive correlation with PGA, SLEDAI, rSLEDAI, and proteinuria (*p* < 0.05, Mann–Whitney *U*-test; *R* > 0.4), with a similar trend being noted for Hemopexin (Fig. 4a; Supplementary Table 3). Within the Asian cohort, urine ALCAM, peroxiredoxin-6, TFPI, and VCAM-1 all significantly correlated with PGA, SLEDAI, and rSLEDAI (*p* < 0.05, Mann–Whitney *U*-test; *R* > 0.4) (Fig. 4a). Several urine proteins displayed a negative correlation with C3/C4 and a positive correlation with anti-DNA, as summarized in Fig. 4a. Although the most discriminatory biomarkers tracked with proteinuria, they are unlikely to be simply the consequence of proteinuria as several other proteins that shared similar molecular weights with these biomarkers were not elevated in the urine (Supplementary Fig. 3). Although the correlation between urine biomarkers and renal pathology LN class was feeble, it should be pointed out that the time interval between the biopsy and urine procurement ranged from 1 month to 20 years; hence, the urine biomarker levels may not be reflective of historical renal pathology data.

We next subjected the 12 assayed urine proteins, ethnicity, and various clinical metrics to an unsupervised Bayesian network analysis, which uses probability distributions to represent the inter-dependencies of all changing variables in a model and how they relate to each other. As one would predict, the three clinical indices monitored, SLEDAI, rSLEDAI, and disease status (active LN vs inactive lupus) were strongly linked to each other, with strong positive correlation (Fig. 4b). Likewise, proteinuria, pyuria, and hematuria were strongly linked to rSLEDAI. The fact that these “ground truth” relationships were correctly identified by the unsupervised Bayesian network algorithm offers internal validation of this probabilistic association approach. This independent analysis identified urine ALCAM as having the strongest impact on “disease status”, and sE-selectin as having the strongest impact on SLEDAI (Fig. 4b), with race being an important confounding factor, as we have already established above. Indeed, these two urine proteins exhibited the largest impact on all other nodes in this network, based on their “node force”, which is proportional to the size of each node.

### Expression of urinary biomarkers within the kidneys in LN

The proteins noted to be elevated in the urine of LN patients could have originated from two potential sources—from the circulating blood, or from within the kidneys. To assess whether the 12 proteins interrogated in this study might also be expressed with the kidneys in LN, we turned to another OMICs platform. Single-cell RNAseq analysis of renal biopsy tissue from LN has recently detailed the expression of all genes within LN kidneys, with imputed cell-of-origin information. Two publically available single-cell RNAseq datasets which contained 1624 kidney single-cell RNAseq profiles from 21 LN patients and 3 healthy controls and 363 cells from 10 patients, respectively, were combined using canonical correlation analysis, and interrogated for expression of the 12 urine biomarkers described above.

**Fig. 5:**
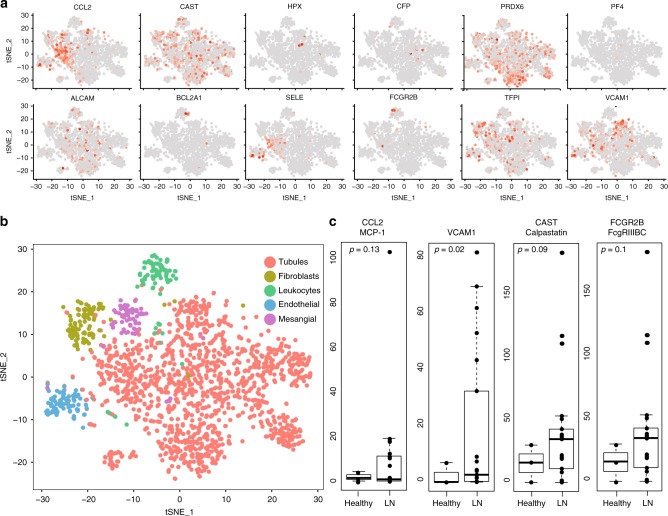
Renal single-cell RNAseq expression of potential urine biomarkers in LN and healthy controls. **a** Feature plots of potential urine biomarkers where each dot is a cell and the color intensity (gray = low, to red = high) indicates expression of the indicated gene within each cell, as deduced by RNAseq data analysis of 21 LN renal tissue samples. CCL2 = MCP-1; CAST = calpastatin; HPX = hemopexin; PRDX6 = peroxiredoxin-6; BCL2A1 = BFL-1; SELE = sE-selectin. **b** tSNE plot of 1624 cells obtained from the renal biopsy of 21 LN patients and 3 healthy controls. Clusters are derived from PCA and graph-based clustering analysis and color-coded based on identified cell type. **c** Student’s *t*-test comparison of expression of CCL2 (MCP-1), VCAM-1, CAST (calpastatin), and FCG2RB between LN patients (*N* = 21) and healthy controls (*N* = 3) within the single-cell RNAseq dataset. The ends of the box represent the upper and lower quartiles, so the box spans the interquartile range. The median is marked by a horizontal line inside the box. The whiskers are the two lines outside the box that indicate 1.5 times the interquartile range from the upper or lower quartile.

Strong intra-renal expression was noted for MCP-1 (CCL2), calpastatin, peroxiredoxin-6, ALCAM, TFPI, and VCAM-1, within LN kidneys (Fig. 5a). Intra-renal endothelial cells in LN expressed sE-selectin, TFPI, VCAM-1, all of which one would have predicted, as well as calpastatin, MCP-1, and peroxiredoxin-6. Strong renal tubular expression of ALCAM, calpastatin, MCP-1, TFPI, VCAM-1, and peroxiredoxin-6 were observed. ALCAM, MCP-1, calpastatin, peroxiredoxin-6, and TFPI were expressed within mesangial cells as well, while infiltrating leukocytes expressed ALCAM, BFL-1, calpastatin, FcGR2B, properdin, and peroxiredoxin-6, as portrayed in Fig. 5a. Despite the limited number of healthy control samples (*N* = 3), renal expression of VCAM-1 was significantly higher in LN, with similar trends being noted for MCP-1, calpastatin, and FcGR2B (Fig. 5b).

## Discussion

Large-scale proteomic screening technologies have revolutionized the study design and workflow for biomarker research. Samples that could previously only be used to measure <100 proteins can now be screened for several thousands of proteins simultaneously using multiple proteomic platforms. Mass spectrometry-based untargeted techniques have been utilized within the context of SLE^7,8,18,19^. Although these platforms do not rely on antigen or ligand binding, the data generated by these untargeted platforms may be noisy and may not reliably detect or measure low-abundance proteins. Targeted assays, like the aptamer-based screening platform, have the potential to accurately detect and quantify low-abundance proteins through protein-ligand interactions. To date, the human proteome project has successfully identified over 30,000 human proteins^20^, and the targeted proteomic platforms available at the time of this study offer coverage of <2000 proteins^10,21^.

The aptamer-based screening platform utilized within this study is, to date, one of the largest proteomic screening platforms available, and its use of aptamer ligands has led to the successful identification of biomarkers in several diseases^10–16^. Importantly, the hits that were identified using this initial screening platform were successfully validated by ELISA, with good correlation ratios being noted between these two platforms (Table 1). Reassuringly, this platform re-identified several urine protein biomarkers that had previously been implicated as biomarkers for LN, including adiponectin, NGAL, and TWEAK; although all of these proteins were elevated in the urine of active LN patients in the aptamer-based screen, they did not rank within the top 50, based on fold-change or *p*-values. This observation offers independent validation of this proteomic screening platform. As elaborated further below, most of the newly identified biomarker candidates have important biological functions relating to inflammation or autoimmunity (Supplementary Table 8). Of importance, this screening platform has been effective in identifying several urine biomarkers of LN that correlate with traditional yardsticks of SLE/LN, but outperform those metrics in terms of sensitivity, NPV, and PPV. Besides representing one of the largest targeted proteomic screen conducted in LN, this study is also unique in highlighting the importance of tailoring the biomarkers to patient ethnicity.

Among African-American patients, the most discriminatory biomarkers that distinguished active LN from inactive disease were urine ALCAM, PF-4, properdin, and VCAM-1. Activated leukocyte cell adhesion molecule (ALCAM/CD166), a transmembrane glycoprotein expressed primarily on activated T cells, is involved in multiple immune and inflammatory responses^22–24^. ALCAM is also a known recruiter for white blood cells in chronic kidney disease^25^, and is upregulated within the glomeruli in animal models of LN^26^. Serum levels of ALCAM correlate with SLE disease activity^27^. In this study, ALCAM surpassed conventional metrics in identifying active LN, with better sensitivity, specificity, PPV and NPV, among African-American patients and Asian patients (but not among Caucasian patients), correlating significantly with PGA, SLEDAI, rSLEDAI, and proteinuria. When all SLE patients were combined, urine ALCAM levels had the strongest bearing on disease activity status, in an unsupervised Bayesian network analysis. Urine ALCAM also emerged as one of the few proteins that distinguished active LN from active non-renal lupus (Fig. 3). Indeed, intra-renal expression within LN kidneys was noted within infiltrating leukocytes, renal tubular cells and mesangial cells, based on RNAseq analysis.

Platelet factor 4 (PF-4, or CXCL4) is CXC chemokine that is chemotactic for monocytes and neutrophils^28,29^. PF-4 has been implicated as a possible urinary biomarker for LN^30^. PF-4 autoantibodies have been found in SLE patients^31^, and studies suggest that these antibodies may correlate to disease activity^32^. Of the 12 urinary proteins examined in this study, urinary PF-4 was one of five proteins that was able to distinguish active LN from inactive disease in all African-American, Caucasian, and Asian patients. Importantly, healthy subjects exhibited minimal levels of urinary PF-4, that are comparable to those seen in inactive LN patients, while active LN patients consistently exhibited marked increases in PF-4 levels (fold-change >5). It was a better disease marker among African-American SLE patients, where its sensitivity, NPV and PPV were among the highest, well surpassing that of anti-DNA and complement.

Properdin (CFP) is a plasma glycoprotein of the complement system that is important in the stabilization of alternative pathway convertases. Properdin was reported to be elevated in the kidney tissue of LN (but not SLE patients without nephritis) but decreased in both serum of SLE patients and in patients with poststreptococcal/membranoproliferative glomerulonephritis^33–35^. Properdin has also been implicated in other inflammatory diseases, including arthritis^36^, and IgA nephropathy^37^. In this study, urine properdin exhibits similar diagnostic characteristics as PF-4, in that it has the ability to distinguish active LN in both ethnic groups of patients, but with the best performance being noted among African-American LN patients, in whom urine properdin exhibited the highest specificity and NPV values in identifying active LN patients, compared to other competing markers and conventional yardsticks. Urine properdin also emerged as one of the few proteins that distinguished active LN from active non-renal lupus (Fig. 3), alluding to its renal specificity.

VCAM-1 (vascular cell adhesion molecule, CD106) is a cell adhesion molecule expressed on endothelial cells. VCAM-1 has been implicated as a biomarker in atherosclerosis^38–40^, and extensively studied in the context of SLE and LN^41–43^. In this study, VCAM-1 was one of top two urine biomarkers that had the potential to distinguish active LN from inactive LN within all ethnic cohorts, with respectable sensitivity, NPV and PPV values, compared to the traditional yardsticks. Urine VCAM-1 correlated significantly with PGA, SLEDAI, rSLEDAI, and proteinuria in all ethnic groups. Urine VCAM-1 also emerged as one of the few proteins that distinguished active LN from active non-renal lupus (Fig. 3), alluding to its renal specificity. Indeed, it is strongly expressed within LN kidneys, based on renal single-cell RNAseq analysis, particularly on endothelial cells, renal tubular cells, and infiltrating leukocytes (Fig. 5).

Among Caucasian patients, the most discriminatory urine proteins were sE-selectin, VCAM-1, BFL-1, and Hemopexin. E-Selectin mediates immune cell adhesion, allowing neutrophils to adhere to vascular endothelial cells. E-selectin has been implicated in several cancers^44–46^, and as a potential biomarker for SLE and LN^47–49^. In this study, while sE-Selectin emerged as the most discriminatory urinary marker within Caucasian LN patients, it was not promising within the African-American cohort. Among Caucasian patients, urine sE-selectin exhibited improved sensitivity, NPV and PPV values, compared to the traditional yardsticks, and correlated significantly with PGA, SLEDAI, rSLEDAI, and proteinuria. Urine sE-selectin also exhibited the strongest bearing on SLEDAI and race, in an unsupervised Bayesian network analysis. Strong intra-renal expression within LN kidneys was also noted within endothelial cells, based on RNAseq analysis. Its diagnostic performance in other ethnic groups clearly warrants further analysis in expanded patient cohorts.

BFL-1 is an anti-apoptosis protein within the BCL2 protein family. BFL-1 is a direct transcription target from the NF-kB pathway and is known to be upregulated by several inflammatory signals, including GM-CSF, IL-1, and CD40. It is believed to play a role in leukocyte activation and survival^50–52^, and has been shown to be overexpressed in B cells of SLE patients^53^. This study implicates that BFL-1 may be a reliable biomarker for LN within Caucasian patients (*p* < 0.0001, Mann–Whitney *U*-test; AUC = 0.808), but baseline levels of BFL-1 are much higher in African-American patients, making it unreliable as a biomarker among these patients (*p* > 0.05, Mann–Whitney *U*-test).

Hemopexin, expressed by hepatocytes, and induced by several inflammatory factors exhibits an anti-inflammatory effect and can induce proteinuria^54–56^. Hemopexin has been implicated as a urinary biomarker for pediatric LN^57^, and is elevated in serum of SLE patients; however, it does not correlate with disease activity/severity^58^. The data presented in this study indicate that urinary hemopexin may differentiate active LN from inactive LN regardless of patient demographics (*p* < 0.05, Mann–Whitney *U*-test; AUC > 0.7), but its potential use among Caucasian patients may be limited due to high background levels even among healthy individuals.

Among Asian patients, the most discriminatory urine proteins were ALCAM, VCAM-1, TFPI, and PF-4. TFPI (tissue factor pathway inhibitor), mainly produced by the endothelial cells and megakaryocytes, is the primary inhibitor of the initiation of blood coagulation^59^. It has been reported that urinary TFPI is elevated in active LN, correlating with rSLEDAI^60,61^.

Renal RNAseq analysis indicated strong expression of MCP-1, calpastatin, peroxiredoxin-6, ALCAM, TFPI, and VCAM-1 within the inflamed kidneys, suggesting that intra-renal cells may be the dominant source of these urinary biomarkers in LN, though this needs to be validated by immunohistochemistry. Given this RNAseq expression data, it is not surprising that these same urine proteins, urine calpastatin, ALCAM, TFPI, and VCAM-1, are also the best at discriminating active LN from active non-renal lupus (Fig. 3). On the other hand, the diseased kidneys in LN may not be the dominant source of BFL-1, hemopexin, properdin or PF-4 in the urine; although this prediction is consistent with the known biology of these molecules, this hypothesis warrants further testing of paired serum and urine samples from the same subjects.

Several aspects of this study could be improved upon. Sample sizes could certainly be expanded to uncover markers that are less discriminatory. Given that this study pursued the validation of only 27 urine proteins, a large number of additional proteins (Supplementary Table 1) await systematic validation in future studies. Since these studies were not performed with urine samples obtained at the time of renal biopsy, we were not able to ascertain the relationship between urine biomarkers and renal pathology features, with one exception. We have recently examined the performance of one of the urine proteins examined here, VCAM-1, in patients from whom concurrent renal biopsies were available^62^. Importantly, urine VCAM-1 surpass C3/C4, anti-DNA, and proteinuria in predicting concurrent renal pathology changes, including endocapillary proliferation, glomerular leukocyte infiltration, fibrinoid necrosis, cellular crescents, and interstitial inflammation, all of which are manifestations of renal pathology activity^62^. Clearly, similar types of analyses need to be executed to assess the biomarker potential of the other proteins described in this manuscript, in predicting concurrent renal pathology. In addition, a longitudinal study is warranted to investigate how these molecules relate to disease pathology and progression over time. Frequently timed collections would be needed in order to identify urine proteins that may herald impending renal flares. Finally, these findings call for mechanistic studies that investigate the pathogenic roles of ALCAM, PF-4, properdin, VCAM-1, and sE-selectin in mediating LN.

## Methods

### Patients, sample collection and sample preparation

Patients were recruited from the University of Texas Southwestern Medical Center’s Renal Clinic, Dallas, TX, Johns Hopkins University School of Medicine, Baltimore, MD or Tuen Mun Hospital, Hong Kong, China. All patients gave informed consent, and this study was approved by the institutional review board of Johns Hopkins University School of Medicine, UT Southwestern Medical Center, Tuen Mun Hospital, and the University of Houston. Patients who had a known history of lupus nephritis were included, as well as patients with SLE but no history of lupus nephritis. Patients with renal failure and pediatric patients were excluded from this study.

Clean-catch midstream urine samples were collected in sterile containers and either placed on ice or refrigerated within 1 h of sample collection. The samples were then aliquoted and stored at −80 °C. At each sample collection visit, the patients were assessed by the attending physician, and the following data were obtained: SLEDAI (Systemic Lupus Erythematosus Disease Activity Index), rSLEDAI (renal SLEDAI), PGA, weight, blood pressure, complete blood count, platelets, erythrocyte sedimentation rate, creatinine, cholesterol, C3, C4, anti-dsDNA, anti-cardiolipin, urinalysis, and urine protein/Cr ratio. For all patients, the hybrid SLEDAI was used, where proteinuria was scored if >0.5 g/24 h. The rSLEDAI summates the renal components of the SLEDAI, including hematuria (>5 red blood cells/high-power field), pyuria (>5 white blood cells/high-power field), proteinuria (>0.5 g/24 h), and urinary casts. Active LN was defined as biopsy-proven LN with rSLEDAI > 0. None of the active LN patients in this study had isolated hematuria or pyuria.

### Aptamer-based screen

Urine samples for the initial aptamer-based screen were obtained from the Renal Clinic of UT Southwestern Medical Center; these samples consisted of 7 patients with active renal disease and SLE (rSLEDAI > 0), 8 patients with SLE but no active renal disease (rSLEDAI = 0, SLEDAI ≤ 6), and 8 healthy controls. Urine samples were morning midstream collections, collected, aliquoted, and stored frozen till the time of assay. All samples were clarified by centrifugation before use. These samples were screened using an aptamer-based screening platform pioneered by Somalogic^10^. This assay uses aptamer–protein interactions to detect proteins within a sample. In the assay, aptamer-coated streptavidin beads are first added to the sample to allow the aptamers to bind to the proteins. Next, the bound proteins are biotinylated, and the aptamer–protein complexes are cleaved from the streptavidin beads. These aptamer–protein complexes are then conjugated to a second streptavidin bead, and aptamers are separated from the proteins. The aptamers are then collected from the sample and quantitated by hybridization to a DNA microarray. The final output is the relative fluorescence unit (RFU) for each protein; these RFU values were then normalized to urinary creatinine and statistically analyzed to determine which proteins were increased in patients with active LN or SLE. The median limit of detection (LOD) of the aptamer-based scan is 1.6 pg/ml. The LOD was determined by spiking proteins into buffer before the assay. The limits of quantitation (LOQ) were established along with the LOD, and the median lower LOQ value is approximately 3-fold higher than the LOD.

### Cross-sectional validation study using ELISA

For the primary cross-sectional study, 95 subjects with African-American and Caucasian ethnicities were included, comprised of 47 inactive SLE patients, 27 active LN patients and 21 healthy controls. These subjects were drawn from two medical centers: Johns Hopkins University School of Medicine, Baltimore, MD and UT Southwestern Medical Center, Dallas, TX. Out of the 27 active LN patients, 23 had proteinuria. The remaining four had hematuria (RBC > 5/HPF) along with pyuria but without proteinuria (urine protein/creatinine <0.5 or 24-hour urine protein <0.5). For these 4 patients, the average time interval between the renal biopsy and urine procurement was 16 months. The second validation cohort included 80 inactive SLE patients, 80 active LN patients, 67 active non-renal lupus patients and 53 healthy controls with Asian ethnicity. Potential biomarkers were validated using commercially available ELISA assays, following manufacturer instructions, in a blinded fashion, where the operator was unaware of the disease group, before data analysis. The vendors and dilutions used are summarized in Supplementary Data. The absolute levels of urine proteins were determined using standard curves run on each ELISA plate, and normalized by urine creatinine. The degree of correlation between the aptamer screening platform and the ELISA-validation platform are presented in Supplementary Data. Briefly, diluted urine samples were added in pre-coated 96-well microplates. After sample incubation, detection antibodies were added, followed by streptavidin-HRP, and substrate. A microplate reader (ELX808 from BioTek Instruments, Winooski, VT) was used to read the optical density at 450 nm. The optimal urine concentration was determined based on a standard curve derived for each molecule, and this is detailed in Supplementary Data. Inter-assay and inter-day variability in these assays were negligible, as summarized in Supplementary Fig. 4.

### RNAseq analysis of renal tissue from LN

Publically available single-cell RNAseq data from patients with biopsy-proven LN and healthy controls was obtained from Immport using accession numbers SDY997 EXP15077 and from the NCBI Short-Read Archive (SRA) under the accession number PRJNA379992^63^. For both datasets, post quality control expression matrices contained both skin and kidney cells and were subsetted to only include kidney cells for downstream analysis, yielding 1624 cells from 21 patients and 3 healthy controls1 and 363 cells from 10 patients^64^. Datasets were combined using canonical correlation analysis using the Seurat package for R as previously described^65^. Graph-based clustering and tSNE was performed on the kidney single-cell profiles using the Seurat package for R^66^. Principal component analysis yielding 12 principal components was used to derive the clusters. Cluster identity was assigned by comparing differentially expressed genes between the clusters to canonical markers. Feature plots were also created using the Seurat package for R^67^. Gene expression comparisons between LN and healthy control tubular cells were performed by first creating a per-patient tubular cell profile and then using the Student’s *t*-test.

### Heatmap, cluster analysis

Data from the aptamer-based screening assay were used to generate heatmaps that cluster proteins with similar expression patterns together. There were two heatmaps generated for this analysis; one heatmap focuses on proteins that were significantly elevated in active LN when compared to inactive LN, and the second focuses on proteins that were significantly elevated in SLE when compared to healthy controls. For both heatmaps, proteins were classified as significantly elevated if they had a *p*-value of <0.05 and a fold-change >2. The data from each group was imported into MATLAB for clustering analysis and heatmap generation. For clustering, proteins were clustered in an unsupervised manner based on Euclidean distance with a maximum cluster size of 20.

### Random forest classification and Bayesian network analysis

Random forest classification analysis, a machine learning algorithm for dimensionality reduction, was executed using 1000 bootstrap sampling iterations, in order to identify the relative importance of each biomarker candidate in disease classification, as measured by the GINI index, using the sklearn.ensembl R package. For the top 20 urine potential biomarkers identified by random forest classification, the fold-change in SLE vs healthy controls, and the fold-change in active LN vs inactive SLE were plotted as a radial plot, using Excel. Bayesian network analysis was performed using the BayesiaLab software (Bayesia, version 7.0.1). The dataset for unsupervised learning included data pertaining to 95 subjects (comprises 47 inactive SLE patients, 27 active LN patients, and 21 healthy controls), including the following parameters: the urine levels of 12 protein biomarkers, race, disease status (active renal vs inactive SLE) and disease features or measures (proteinuria, pyuria, hematuria, SLEDAI, rSLEDAI, PGA, and eGFR). Continuous data were discretized into 3 bins using the R2-GenOpt algorithm and the Maximum Weight Spanning Tree algorithm was used for unsupervised learning of the network. Under these conditions, all parameters were connected in the generated network model.

### Data analysis

Biomarker data were plotted and analyzed using either GraphPad Prism 5 (GraphPad, San Diego, CA), Microsoft Excel 2016 (Microsoft Corporation), MATLAB R2016 (Natick, MA), or available packages t R 3.4.1 (R Foundation for Statistical Computing, Vienna, Austria). Biomarker group comparisons were analyzed using the Mann–Whitney *U*-test as several datasets were not normally distributed. Statistical *p*-values and *q*-values (*p*-values adjusted for false discovery rate, for multiple testing correction) were computed for each biomarker. The Spearman method was used for the correlation analysis, and the Kruskal–Wallis test was used for multiple comparisons.

A value of 1 was added to all biomarker measurements, then log-transformed to base 2. To examine the relationship between an individual biomarker and outcomes, we performed logistic regression models for active lupus nephritis, and linear regression models for continuous outcomes including PGA scores, eGFR, and rSLEDAI. For each outcome, three models were ran to control for race, race and age, race and prednisone administration. For each model, *q*-values (*p*-values adjusted for false discovery rate) were computed for each biomarker. We calculated the area under the ROC (receiver operating characteristic) curve for models including a biomarker and race with active lupus as the outcome. We used LASSO (least absolute shrinkage and selection operator) to select the subset of biomarkers and patients’ characteristics that is most predictive of active lupus nephritis status. All measurements were standardized before running LASSO. Ten-fold cross validation was carried out to select the tuning parameter lambda. The largest value of lambda such that error is within 1 standard error of the minimum was selected to fit LASSO. Then active status was predicted based on the estimated LASSO coefficients. ROC curve was plotted with area under the curve to demonstrate the discriminative power of the group of variables selected by LASSO. Race was purposely kept in the model throughout cross validation and LASSO fitting process. We examined two models with LASSO: Model 1 includes all biomarkers and race, and Model 2 includes all biomarkers and race, age and prednisone administration. All the calculations were done in R 3.4.1. *q*-values were computed using the qvalue package. ROC curves were plotted and areas under the ROC curve were calculated using the pROC package. Cross validation was conducted and LASSO coefficients were estimated using the glmnet package.

### Reporting summary

Further information on research design is available in the Nature Research Reporting Summary linked to this article.

## Supplementary information

Supplementary Information

Reporting Summary

## Data Availability

Source data are provided as a Source Data files. The source data are also freely available at: http://hoc.bme.uh.edu/services/targeted-proteomics/examples-of-proteomic-screens/. Public single-cell RNAseq datasets used for the analysis are from Immport batabase, accession numbers SDY997 EXP15077, and from the NCBI Short-Read Archive (SRA), accession number PRJNA379992^63^.

## Source data

Source data

## Code availability

Various R packages were used, and no unique code was written for this work.
