# Author Correction: Comprehensive aptamer-based screening identifies a spectrum of urinary biomarkers of lupus nephritis across ethnicities

**DOI:** 10.1038/s41467-020-16944-9

**Published:** 2020-06-16

**Authors:** Samantha Stanley, Kamala Vanarsa, Samar Soliman, Deena Habazi, Claudia Pedroza, Gabriel Gidley, Ting Zhang, Shree Mohan, Evan Der, Hemant Suryawanshi, Thomas Tuschl, Jill Buyon, Chaim Putterman, Chi Chiu Mok, Michelle Petri, Ramesh Saxena, Chandra Mohan

**Affiliations:** 1 Department Biomedical Engineering, University of Houston, Houston, TX USA; 2 Rheumatology and Rehabilitation Department, Faculty of Medicine, Minia University, Minya, Egypt; 3 Center for Clinical Research and Evidence-Based Medicine, University of Texas Health Science Center at Houston, Houston, TX USA; 4 Department of Rheumatology, Albert Einstein College of Medicine, Bronx, NY USA; 5 Department of Molecular Biology, Rockefeller University, New York, NY USA; 6 Department of Rheumatology, New York University, New York, NY USA; 7 Azrieli Faculty of Medicine, Bar-Ilan University, Zefat, Israel; 8 Research Institute, Galilee Medical Center, Nahariya, Israel; 9 Department of Medicine, Tuen Mun Hospital, New Territories, Hong Kong, China; 10 Division of Rheumatology, Johns Hopkins University School of Medicine, Baltimore, MD USA; 11 University Hospital Kidney & Liver Clinic, University of Texas Southwestern Medical Center, Dallas, TX USA

**Keywords:** Proteomics, Diagnostic markers, Lupus nephritis, Population screening

Correction to: *Nature Communications* 10.1038/s41467-020-15986-3, published online 4 May 2020.

The original version of this Article contained an error in the author affiliations.

The affiliations of Chaim Putterman with ‘Azrieli Faculty of Medicine, Bar-Ilan University, Zefat, Israel’ and ‘Research Institute, Galilee Medical Center, Nahariya, Israel’ were inadvertently omitted.

This has now been corrected in both the PDF and HTML versions of the Article.

