# Supplementary material for: Comprehensive aptamer-based screening identifies a spectrum of urinary biomarkers of lupus nephritis across ethnicities
Source: Nat Commun. 2020 May 4;11:2197. doi: 10.1038/s41467-020-15986-3 (PMC7198599; doi:10.1038/s41467-020-15986-3)
Supplement: Supplementary file 1 — Supplementary Information [file 41467_2020_15986_MOESM1_ESM.docx]

**Comprehensive aptamer-based screening identifies a spectrum of urinary biomarker**

**of lupus nephritis across ethnicities**

Stanley et al.

| **Supplementary Table 1: Top 50 LN Urinary Biomarkers from Aptamer-Based Screening^1^** | | | | | | |
| --- | --- | --- | --- | --- | --- | --- |
| **Protein** | **Urine protein level (Cr-normalized RFU)** | | | | **Fold Change^2^** | |
|  | **Mean (Median)** | | | |  |  |
|  | **Healthy** | **Inactive SLE** | **Active LN** | **All SLE** | **SLE/ Healthy** | **Active LN/ InactiveSLE** |
|  | **N = 8** | **N = 8** | **N = 7** | **N = 15** |  |  |
| 40s Ribos. SA | 47 (45) | 116 (62) | 1498 (914) | 761 (344) | 16.25* | *12.94** |
| Activin A | 16 (16) | 22 (16) | 383 (371) | 190 (46) | 11.9* | *17.43** |
| AIF1 | 225 (153) | 294 (277) | 4926 (3814) | 2456 (433) | 10.91* | *16.74** |
| ALCAM | 224 (237) | 616 (326) | 4055 (4990) | 2221 (789) | 9.91 | 6.59* |
| BAFF Receptor | 55 (45) | 108 (45) | 732 (702) | 399 (254) | 7.28* | *6.75*** |
| BFL1 | 98 (58) | 220 (111) | 2622 (1634) | 1341 (539) | 13.67* | *11.94*** |
| Complement C4 | 607 (402) | 3778 (1240) | 29577 (17987) | 15817 (7193) | 26.04* | *7.83*** |
| Complement C6 | 331 (310) | 1272 (656) | 22294 (10459) | 11082 (5314) | 33.5* | *17.53*** |
| Cadherin-5/VE | 116 (106) | 413 (150) | 5451 (4057) | 2764 (1266) | 23.85* | *13.19*** |
| Calpastatin | 1047 (391) | 2745 (1502) | 18267 (13332) | 9989 (5555) | 9.54** | *6.66*** |
| CAMK1 | 180 (180) | 349 (155) | 3220 (2446) | 1688 (670) | 9.4 | *9.24*** |
| CTAP-III | 332 (277) | 1251 (906) | 37089 (24883) | 17975 (4381) | 54.21** | *29.65*** |
| Cytochrome P450 | 362 (382) | 603 (355) | 4007 (3219) | 2192 (1120) | 6.05* | *6.64*** |
| FCGR2A/B | 100 (58) | 130 (168) | 1282 (584) | 668 (203) | 6.67 | *9.85** |
| Fibronectin | 2555 (1599) | 3217 (1969) | 25793 (16606) | 13752 (10456) | 5.38* | *8.02*** |
| GOT1 | 270 (280) | 623 (290) | 4011 (3041) | 2204 (930) | 8.15 | *6.44** |
| Hemopexin | 346 (297) | 1379 (771) | 13163 (7272) | 6878 (3507) | 19.89* | *9.55*** |
| HGFA | 1002 (764) | 5211 (1328) | 35091 (28877) | 19155 (8852) | 19.13 | *6.73** |
| Histone H1.2 | 50 (10) | 96 (83) | 1259 (1058) | 639 (257) | 12.69* | *13.06*** |
| HPG (PTGR1) | 251 (244) | 767 (365) | 6856 (7585) | 3609 (1822) | 14.36* | 8.94** |
| HSP 60 | 150 (111) | 269 (157) | 2718 (3237) | 1411 (377) | 9.42 | *10.12** |
| IgM | 1071 (575) | 1498 (1320) | 21595 (18564) | 10877 (1911) | 10.15 | 14.42* |
| IL-12 RB2 | 18 (15) | 27 (19) | 223 (214) | 119 (75) | 6.69* | *8.14*** |
| IL-16 | 61 (32) | 60 (53) | 1845 (2133) | 893 (94) | 14.74* | *30.9*** |
| LCMT1 | 141 (145) | 221 (150) | 1457 (913) | 798 (511) | 5.65* | *6.6*** |
| LY86 | 1823 (1618) | 2591 (981) | 19301 (15806) | 10389 (4993) | 5.7 | 7.45** |
| MCP-1 | 57 (41) | 116 (76) | 1061 (1040) | 557 (171) | 9.84* | *9.14*** |
| MIF | 89 (86) | 146 (149) | 1086 (1201) | 585 (209) | 6.55* | 7.43** |
| NAP-2 | 112 (93) | 307 (226) | 9488 (5359) | 4591 (1034) | 40.83** | *30.91*** |
| p27Kip1 | 18 (14) | 27 (19) | 215 (238) | 115 (66) | 6.41* | *7.88*** |
| PAFAH beta | 177 (122) | 271 (146) | 1732 (1542) | 953 (644) | 5.38* | *6.39*** |
| Peroxiredoxin-6 | 4160 (4415) | 7447 (5285) | 54287 (41091) | 29306 (12905) | 7.05* | 7.29** |
| PF-4 | 261 (253) | 995 (480) | 45741 (35395) | 21877 (3035) | 83.91* | 45.96** |
| PFD5 | 28 (30) | 57 (33) | 585 (386) | 303 (91) | 10.96 | *10.3** |
| PGP9.5 | 616 (161) | 839 (250) | 10611 (6449) | 5399 (1993) | 8.77* | 12.65** |
| PGK1 | 120 (115) | 427 (98) | 3739 (4315) | 1972 (950) | 16.38 | *8.77*** |
| Properdin | 805 (268) | 1307 (933) | 13272 (7442) | 6890 (3709) | 8.56* | *10.16*** |
| PSME1 | 196 (171) | 530 (383) | 4335 (3280) | 2306 (1034) | 11.74* | *8.17** |
| RUXF | 31 (24) | 60 (42) | 404 (493) | 220 (121) | 7.19* | 6.78** |
| sE-Selectin | 230 (90) | 367 (167) | 3033 (3057) | 1611 (305) | 7* | *8.26*** |
| SOD | 39 (32) | 153 (44) | 3963 (5174) | 1931 (313) | 49.27* | *25.91*** |
| SP-D | 650 (104) | 1923 (1388) | 15505 (19170) | 8261 (3575) | 12.72 | 8.06* |
| Tenascin | 425 (364) | 541 (478) | 5326 (4916) | 2774 (867) | 6.53 | *9.85** |
| TFPI | 1310 (1250) | 1298 (1003) | 8426 (6911) | 4624 (2438) | 3.53 | 6.49** |
| TIMP-1 | 598 (439) | 854 (846) | 8899 (8580) | 4608 (1476) | 7.7 | *10.42*** |
| TLR2 | 30 (24) | 64 (37) | 420 (380) | 230 (164) | 7.66* | *6.59*** |
| TWEAKR | 14 (11) | 31 (26) | 204 (219) | 112 (53) | 8.11* | *6.67** |
| TXD12 | 101 (99) | 172 (131) | 1381 (1147) | 736 (434) | 7.27* | *8.04*** |
| VCAM-1 | 75 (65) | 122 (90) | 1885 (1959) | 945 (201) | 12.61* | 15.4** |
| VEGF sR3 | 27 (25) | 41 (34) | 368 (159) | 193 (74) | 7.21* | *9.07*** |
|  |  |  |  |  |  |  |

1: Listed are the top 50 urine proteins, ranked based on fold-change in the active LN vs inactive SLE comparison, starting with all proteins that were significantly elevated in both these comparisons: all_SLE vs healthy urine and active LN vs inactive disease. All biomarker levels are shown normalized to urine protein.

2: Indicated are the statistical significance p-values as determined by Mann Whitney U test (*, p <0.05; **, p <0.01; ***, p<0.001). If the elevation of the molecule in active LN urine was still significant after multiple testing correction (q <0.05), this is indicated by an italicized fold change value.

| **Supplementary Table 2. Validation of the top 50 urine proteins significantly elevated in active LN urine, on the aptamer-based screen** | | | |
| --- | --- | --- | --- |
| Protein | ELISA Manufacturer* | Urine Dilution | Notes |
| 40s Ribos. SA | MyBioSource | N/A | Protein was not detectable by ELISA |
| Activin A | RayBioTech | 1:02 | On preliminary ELISA validation, levels in active LN were not higher than controls^1^ |
| AIF1 | LifeSpan BioSciences, Inc. | N/A | Commercial ELISA Kit did not work. |
| **ALCAM** | **R&D Systems** | **1:50** | **Selected for further validation in an extended cross-sectional cohort^2^** |
| BAFF Receptor | LifeSpan BioSciences, Inc. | 1:02 | On preliminary ELISA validation, levels in active LN were not higher than controls^1^ |
| **BFL1** | **MyBioSource** | **1:20** | **Selected for further validation in an extended cross-sectional cohort^2^** |
| C4 | Abnova | 1:02 | On preliminary ELISA validation, levels in active LN were not higher than controls^1^ |
| C6 | Innovative Research | 1:25 | On preliminary ELISA validation, levels in active LN were not higher than controls^1^ |
| **Calpastatin** | **MyBioSource** | **Neat** | **Selected for further validation in an extended cross-sectional cohort^2^** |
| CAMK1 | ND (see Notes)* | N/A | On the screen, results correlate >90% with another selected protein (IL-16) |
| CTAP-III | ND (see Notes) | N/A | On the screen, levels correlate >90% with other selected protein (NAP2) |
| Cytochrome P450 | ND (see Notes) | N/A | On the screen, levels correlate >90% with other selected protein (IL-16) |
| **E-Selectin** | **R&D Systems** | **1:05** | **Selected for further validation in an extended cross-sectional cohort^2^** |
| **FcγRIIBC** | **RayBioTech** | **1:02** | **Selected for further validation in an extended cross-sectional cohort^2^** |
| Fibrinogen | Immunology Consultants Lab | 1:50 | On preliminary ELISA validation, levels in active LN were not higher than controls^1^ |
| Fibronectin | R&D Systems | 1:05 | On preliminary ELISA validation, levels in active LN were not higher than controls^1^ |
| GOT1 | ND (see Notes) | N/A | On the screen, levels correlate >90% with other selected protein (IL-16) |
| **Hemopexin** | **Immunology Consultants** | **1:50** | **Selected for further validation in an extended cross-sectional cohort^2^** |
| HGFA | ND (see Notes) | N/A | On the screen, levels correlate >90% with other selected protein (properdin) |
| Histone H1.2 | ND (see Notes) | N/A | On the screen, levels correlate >90% with other selected protein (BAFF-R) |
| HSP60 | R&D Systems | 1:02 | On preliminary ELISA validation, levels in active LN were not higher than controls^1^ |
| IgM | ND (see Notes) | N/A | Not pursued, as extensive literature already exists. |
| IL-12Rb2 | ND (see Notes) | N/A | Not pursued as the intensity units on the aptamer-screen were <1000 RFU |
| IL-16 | R&D Systems | 1:01 | On preliminary ELISA validation, levels in active LN were not higher than controls^1^ |
| LCMT1 | ND (see Notes) | N/A | On the screen, levels correlate >90% with other selected protein (IL-16) |
| LY86 | MyBioSource | 1:01 | On preliminary ELISA validation, levels in active LN were not higher than controls^1^ |
| **MCP-1** | **R&D Systems** | **1:10** | **Selected for further validation in an extended cross-sectional cohort^2^** |
| MIF | ND (see Notes) | N/A | Not pursued, as extensive literature already exists. |
| NAP-2 | R&D Systems | 1:10 | On preliminary ELISA validation, levels in active LN were not higher than controls^1^ |
| p27Kip1 | ND (see Notes) | N/A | Not pursued as the intensity units on the aptamer-screen were <1000 RFU |
| PAFAHβ1 | ND (see Notes) | N/A | On the screen, levels correlate >90% with other selected protein (IL-16) |
| **Peroxiredoxin 6** | **Abcam** | **Neat** | **Selected for further validation in an extended cross-sectional cohort^2^** |
| **PF-4** | **R&D Systems** | **1:05** | **Selected for further validation in an extended cross-sectional cohort^2^** |
| PFD5 | ND (see Notes) | N/A | Not pursued as the intensity units on the aptamer-screen were <1000 RFU |
| PGP9.5 | ND (see Notes) | N/A | On the screen, levels correlate >90% with other selected protein (BFL1, Calpastatin) |
| PGK1 | Cloud-Clone Corp | 1:02 | On preliminary ELISA validation, levels in active LN were not higher than controls^1^ |
| **Properdin** | **LifeSpan BioSciences, Inc.** | **1:02** | **Selected for further validation in an extended cross-sectional cohort^2^** |
| PSME1 | ND (see Notes) | N/A | On the screen, levels correlate >90% with other selected protein (BFL1) |
| RUXF | ND (see Notes) | N/A | Not pursued as the intensity units on the aptamer-screen were <1000 RFU |
| SOD | R&D Systems | N/A | On preliminary ELISA validation, levels in active LN were not higher than controls^1^ |
| SP-D | ND (see Notes) | N/A | On the screen, levels correlate >90% with other selected protein (ALCAM) |
| Tenascin-XB | LifeSpan BioSciences, Inc. | 1:10 | On preliminary ELISA validation, levels in active LN were not higher than controls^1^ |
| **TFPI** | **R&D Systems** | **1:02** | **Selected for further validation in an extended cross-sectional cohort^2^** |
| TIMP1 | R&D Systems | 1:20 | On preliminary ELISA validation, levels in active LN were not higher than controls^1^ |
| TLR2 | ND (see Notes) | N/A | Not pursued as the intensity units on the aptamer-screen were <1000 RFU |
| TWEAKR | ND (see Notes) | N/A | Not pursued as the intensity units on the aptamer-screen were <1000 RFU |
| TXD-12 | ND (see Notes) | N/A | On the screen, levels correlate >90% with other selected protein (BFL1, Calpastatin) |
| **VCAM-1** | **R&D Systems** | **1:10** | **Selected for further validation in an extended cross-sectional cohort^2^** |
| VE-Cadherin | R&D Systems | 1:05 | On preliminary ELISA validation, levels in active LN were not higher than controls^1^ |
| VEGFsR3 | ND (see Notes) | N/A | Not pursued as the intensity units on the aptamer-screen were <1000 RFU |
| * ELISA kits were purchased for 29 of the listed proteins. The rest were not pursued for the reasons listed under Notes. | | | |
| 1: In a pilot ELISA test using a cohort that was independent of the cohort used for the initial aptamer-based screen, this marker was not significantly higher in active LN (N = 12), compared to inactive SLE (N =12) and healthy control (N = 12). Hence, these proteins were not pursued further. | | | |
| 2: In a pilot ELISA test using a cohort that was independent of the cohort used for the initial aptamer-based screen, this marker was significantly higher in active LN (N = 12), compared to inactive SLE (N =12) and healthy control (N = 12). Hence, these markers were further ELISA-tested in an extended cohort of LN/SLE patients, as detailed in Fig. 2 and Table 1. | | | |
|  | | | |

|  | **Supplementary Table 3. Urine Cr-normalized Biomarker Levels in cross-sectional cohort, parsed by ethnicity** | | | | | | | | | |  |  |
| --- | --- | --- | --- | --- | --- | --- | --- | --- | --- | --- | --- | --- |
|  | **Urine Biomarker Levels in African-American SLE patients and controls** | | | | | | | | | |  |  |
|  |  |  |  |  | **Fold Change^2^** | |  | |  |  |  |  |
| **Urine Protein** | **Urine protein, pg/mg, Mean (Median)** | | | | **Active/** | **SLE/** | **Comparison of active LN versus inactive SLE^2^** | | | | | |
|  | **Healthy** | **Inactive SLE** | **Active LN** | **All SLE** | **Inactive** | **Healthy** | **Cut-off** | **ROC AUC** | **Sensitivity** | **Specificity** | **NPV** | **PPV** |
| ALCAM | 32 (0) | 3398 (1631) | 8483 (8761) | 5555 (2276) | 2.5** | 173.2**** | 4875 | 0.84**** | 0.79 | 0.95 | 0.92 | 0.86 |
| BFL1 | 10 (8) | 40 (4) | 37 (35) | 39 (18) | 0.9 | 3.9 | 25 | 0.62 | 0.64 | 0.74 | 0.64 | 0.74 |
| Calpastatin | 23 (0) | 82 (0) | 1667 (38) | 726 (0) | 20.3** | 31.6 | 808 | 0.72** | 0.50 | 0.95 | 0.88 | 0.72 |
| FcgRIIBC | 113 (0) | 351 (269) | 1411 (856) | 761 (374) | 4** | 6.8** | 442 | 0.82**** | 0.79 | 0.79 | 0.73 | 0.83 |
| Hemopexin | 317K(153K) | 1373K(729K) | 2491K(1287K) | 1827K(1008K) | 1.8* | 5.8** | 871586 | 0.73** | 0.86 | 0.58 | 0.60 | 0.85 |
| MCP-1 | 76 (17) | 249 (39) | 697 (310) | 431 (179) | 2.8** | 5.7* | 148 | 0.79*** | 0.93 | 0.68 | 0.68 | 0.93 |
| Peroxiredoxin6 | 24 (0) | 123 (0) | 895 (0) | 436 (0) | 7.3 | 18.2 | 1 | 0.64* | 0.50 | 0.79 | 0.64 | 0.68 |
| PF-4 | 4 (0) | 135 (0) | 1098 (660) | 508 (0) | 8.1*** | 123* | 39 | 0.88**** | 0.93 | 0.79 | 0.77 | 0.94 |
| Properdin | 3217 (0) | 11K (0) | 38K (16K) | 21K (0) | 3.5**** | 6.8 | 5635 | 0.85**** | 0.86 | 0.90 | 0.86 | 0.90 |
| sE-Selectin | 9 (0) | 211 (157) | 298 (185) | 246 (171) | 1.4 | 28*** | 423 | 0.56 | 0.36 | 0.90 | 0.71 | 0.65 |
| TFPI | 10 (5) | 85 (67) | 195 (161) | 128 (69) | 2.3* | 12.8*** | 175 | 0.71* | 0.57 | 0.84 | 0.73 | 0.73 |
| VCAM-1 | 184K (0) | 11K (2228) | 43K (37K) | 24K (11K) | 3.9*** | 0.1 | 10324 | 0.87**** | 0.93 | 0.74 | 0.72 | 0.93 |
| *C3* | N/A | 106 (98) | 96 (97) | 101 (97) | 0.9 | N/A | 96 | 0.41 | 0.57 | 0.47 | 0.44 | 0.60 |
| *C4* | N/A | 21 (20) | 23 (23) | 22 (22) | 1.1 | N/A | 30 | 0.59 | 0.36 | 0.90 | 0.71 | 0.65 |
| *anti-DNA* | N/A | 91.8 (0) | 121 (20) | 104 (0) | 1.3 | N/A | 640 | 0.52 | 0.14 | 0.95 | 0.67 | 0.60 |
|  | **Urine Biomarker Levels in Caucasian SLE patients and controls** | | | | | | | | | |  |  |
| ALCAM | 234 (67) | 1785 (527) | 5906 (1815) | 3092 (1051) | 3.3 | 13.2** | 1588 | 0.66 | 0.69 | 0.75 | 0.56 | 0.84 |
| BFL1 | 0 (0) | 0 (0) | 67 (14) | 21 (0) | N/A** | N/A | 13 | 0.81**** | 0.62 | 1.00 | 1.00 | 0.85 |
| Calpastatin | 0 (0) | 0 (0) | 2506 (325) | 795 (0) | N/A | N/A | 325 | 0.75** | 0.54 | 1.00 | 1.00 | 0.82 |
| FcgRIIBC | 283 (208) | 209 (0) | 279 (98) | 230 (0) | 1.3 | 0.8 | 196 | 0.58 | 0.54 | 0.75 | 0.50 | 0.78 |
| Hemopexin | 788K(657K) | 959K (1K) | 845K (520K) | 923K (263K) | 0.9** | 1.2 | 200487 | 0.80**** | 1.00 | 0.64 | 0.57 | 1.00 |
| MCP-1 | 178 (115) | 322 (180) | 991 (446) | 534 (210) | 3.1 | 3 | 424 | 0.65 | 0.62 | 0.75 | 0.53 | 0.81 |
| Peroxiredoxin6 | 167 (0) | 20 (0) | 0 (0) | 14 (0) | 0 | 0.1 | 2 | 0.50 | 0.08 | 0.93 | 0.33 | 0.68 |
| PF-4 | 0 (0) | 25 (0) | 133 (112) | 57 (0) | 5.3* | N/A | 133 | 0.72* | 0.54 | 0.96 | 0.88 | 0.82 |
| Properdin | 791 (595) | 1542 (0) | 28K (2598) | 9331 (0) | 18.2 | 11.8 | 2062 | 0.71* | 0.62 | 0.89 | 0.73 | 0.83 |
| sE-Selectin | 1 (0) | 1 (0) | 137 (83) | 42 (0) | 137*** | 42 | 3 | 0.87**** | 0.77 | 0.96 | 0.91 | 0.90 |
| TFPI | 37 (0) | 97 (89) | 153 (159) | 114 (90) | 1.6 | 3.1* | 150 | 0.60 | 0.62 | 0.75 | 0.53 | 0.81 |
| VCAM-1 | 8672 (692) | 15K (2355) | 21K (15K) | 16K (4K) | 1.4*** | 1.8 | 4908 | 0.84**** | 0.92 | 0.71 | 0.60 | 0.95 |
| *C3* | N/A | 110 (105) | 96 (81) | 106 (105) | 0.9 | N/A | 146 | 0.38 | 0.31 | 0.93 | 0.67 | 0.74 |
| *C4* | N/A | 21 (20) | 18 (13) | 21 (20) | 0.9 | N/A | 42 | 0.39 | 0.08 | 1.00 | 1.00 | 0.70 |
| *anti-DNA* | N/A | 13 (0) | 187 (40) | 63 (0) | 14.4 | N/A | 40 | 0.74** | 0.54 | 0.96 | 0.88 | 0.82 |
|  |  |  |  |  |  |  |  |  |  |  |  |  |

*Note:* All biomarker levels are shown normalized to urine protein. Indicated are the statistical significance p-values as determined by Mann Whitney U test (*, p <0.05; **, p <0.01; ***, p<0.001, ****, p<0.0001).

| **Supplementary Table 4**: Sample size needed to discriminate active LN from inactive SLE, based on power calculations using the aptamer-based screen and primary ELISA validation data | | |
| --- | --- | --- |
|  | **Sample size needed for 80% power at** α **= 0.05** | |
| Protein | **based on Aptamer screen ^1^** | **based on primary ELISA^2^** |
| ALCAM | 7 | 20 |
| BFL1 | 8 | 30 |
| Calpastatin | 6 | 13 |
| FCG2A/B | 10 | 23 |
| Hemopexin | 9 | 260 |
| MCP-1 | 6 | 15 |
| Peroxiredoxin-6 | 8 | 74 |
| PF-4 | 6 | 20 |
| Properdin | 8 | 24 |
| sE-Selectin | 6 | 36 |
| TFPI | 5 | 57 |
| VCAM-1 | 6 | 56 |
| *1: Estimated based on the observed Cr-normalized values for the 12 indicated proteins, in the initial aptamer-based screen.*  *2: Estimated based on the Cr-normalized values for the 12 indicated proteins, by ELISA, in the primary validation cohort comprised of AA and Caucasian patients* | | |

| **Supplementary Table 5. The second (Chinese) cohort used for validation studies** | | | | |
| --- | --- | --- | --- | --- |
|  | **Active LN** | **Active NR** | **Inactive SLE** | **Healthy Control** |
|  | N = 80 | N = 67 | N = 80 | N = 53 |
| Age (years) | 37.8 ± 14 | 35.1 ± 14 | 44 ± 12.4 | 25.8 ± 3.9 |
| Female, no. (%) | 75 (93.8%) | 62 (92.5%) | 77 (96.3%) | 51 (96.2%) |
| SLEDAI, median (interquartile) | 15.5 (12-18.3) | 9 (6-13) | 2 (0-4) | N/A |
| rSLEDAI, median (interquartile) | 8 (8-12) | 0 (0-0) | 0 (0-0) | N/A |
| PGA, median (interquartile) | 2.2 (2-3) | 1.9 (1.6-2.2) | 0.1 (0.1-0.3) | N/A |
| Protein-to-Creatinine Ratio (mg/mg) | 3.4 ± 3.2 | N/A | N/A | N/A |
| Positive anti-dsDNA/ total tested | 80 / 80 | 67 / 67 | 79 / 80 | N/A |
| Hypocomplementemia/ total tested | 80 / 80 | 67 / 67 | 79 / 80 | N/A |
| **Concurrent medicine use, n (%)** | | | | |
| Prednisone | 72 (90%) | 62 (92.5%) | 65 (81.3%) | N/A |
| Immunosuppressants | 73 (91.2%) | 48 (71.6%) | 54 (67.5%) | N/A |
| Plaquenil | 65 (81.2%) | 62 (92.5%) | 61 (76.2%) | N/A |

| **Supplementary Table 6.** **Sensitivities and specificities of biomarkers in the Chinese cohort using the same cutoff values in the primary cohort** | | |
| --- | --- | --- |
| **Urine Protein** | **Sensitivity** | **Specificity** |
| ALCAM | 0.9375 | 0.7 |
| Calpastatin | 0.575 | 0.825 |
| Hemopexin | 0.925 | 0.9875 |
| Peroxiredoxin-6 | 0.6125 | 0.7625 |
| PF-4 | 0.7125 | 0.95 |
| Properdin | 0.6 | 0.8375 |
| TFPI | 0.8875 | 0.75 |
| VCAM-1 | 0.9625 | 0.65 |
| C3 | 0 | 0.8625 |
| C4 | 0.0125 | 0.925 |
| *anti-DNA* | 0.8625 | 0.425 |

| **Supplementary Table 7. Association of urine biomarkers with disease activity indices after adjusting for confounding factors** | | | | | | | | | |
| --- | --- | --- | --- | --- | --- | --- | --- | --- | --- |
| **Association of urine protein with rSLEDAI** | | | | | | | | | |
| **Urine Protein** | **Race Co-Factor** | | | **Race + Age Co-Factors** | | | **Race + Prednisone Co-Factors** | | |
|  | **Beta ± Std Error** | **P-Value** | **Q-Value** | **Beta ± Std Error** | **P-Value** | **Q-Value** | **Beta ± Std Error** | **P-Value** | **Q-Value** |
| ALCAM | 0.38 ± 0.138 | ** | * | 0.374 ± 0.138 | ** | * | 0.305 ± 0.139 | * | * |
| BFL-1 | 0.773 ± 0.177 | *** | *** | 0.756 ± 0.18 | *** | *** | 0.695 ± 0.177 | *** | *** |
| Calpastatin | 0.539 ± 0.093 | *** | *** | 0.533 ± 0.094 | *** | *** | 0.503 ± 0.093 | *** | *** |
| FcgRIIBC | 0.217 ± 0.123 | ns | ns | 0.2 ± 0.125 | ns | ns | 0.204 ± 0.12 | ns | ns |
| Hemopexin | 0.389 ± 0.098 | *** | *** | 0.401 ± 0.106 | *** | *** | 0.34 ± 0.101 | ** | ** |
| MCP-1 | 0.487 ± 0.15 | ** | ** | 0.477 ± 0.15 | ** | ** | 0.441 ± 0.147 | ** | ** |
| Peroxiredoxin-6 | 0.023 ± 0.176 | ns | ns | 0.017 ± 0.176 | ns | ns | 0.082 ± 0.17 | ns | ns |
| PF-4 | 0.563 ± 0.11 | *** | *** | 0.567 ± 0.11 | *** | *** | 0.532 ± 0.109 | *** | *** |
| Properdin | 0.352 ± 0.064 | *** | *** | 0.349 ± 0.065 | *** | *** | 0.329 ± 0.065 | *** | *** |
| sE-Selectin | 0.431 ± 0.154 | ** | * | 0.42 ± 0.155 | ** | * | 0.399 ± 0.151 | * | * |
| TFPI | 0.301 ± 0.216 | ns | ns | 0.291 ± 0.218 | ns | ns | 0.215 ± 0.216 | ns | ns |
| VCAM-1 | 0.356 ± 0.083 | *** | *** | 0.355 ± 0.085 | *** | *** | 0.324 ± 0.084 | *** | *** |
| **Association of urine protein with PGA** | | | | | | | | | |
| **Urine Protein** | **Race Co-Factor** | | | **Race + Age Co-Factors** | | | **Race + Prednisone Co-Factors** | | |
|  | **Beta ± Std Error** | **P-Value** | **Q-Value** | **Beta ± Std Error** | **P-Value** | **Q-Value** | **Beta ± Std Error** | **P-Value** | **Q-Value** |
| ALCAM | 0.045 ± 0.026 | ns | ns | 0.043 ± 0.026 | ns | ns | 0.025 ± 0.026 | ns | ns |
| BFL-1 | 0.1 ± 0.035 | ** | * | 0.091 ± 0.035 | * | * | 0.078 ± 0.034 | * | * |
| Calpastatin | 0.055 ± 0.02 | ** | * | 0.052 ± 0.02 | * | * | 0.043 ± 0.019 | * | * |
| FcgRIIBC | 0.06 ± 0.022 | ** | * | 0.055 ± 0.022 | * | * | 0.057 ± 0.021 | ** | * |
| Hemopexin | 0.071 ± 0.018 | *** | *** | 0.067 ± 0.02 | ** | ** | 0.058 ± 0.018 | ** | ** |
| MCP-1 | 0.077 ± 0.028 | ** | * | 0.073 ± 0.028 | * | * | 0.066 ± 0.027 | * | * |
| Peroxiredoxin-6 | 0.03 ± 0.032 | ns | ns | 0.028 ± 0.032 | ns | ns | 0.044 ± 0.03 | ns | ns |
| PF-4 | 0.081 ± 0.022 | *** | ** | 0.082 ± 0.021 | *** | ** | 0.072 ± 0.021 | *** | ** |
| Properdin | 0.055 ± 0.013 | *** | *** | 0.053 ± 0.013 | *** | *** | 0.048 ± 0.013 | *** | ** |
| sE-Selectin | 0.079 ± 0.028 | ** | * | 0.075 ± 0.027 | ** | * | 0.072 ± 0.027 | ** | * |
| TFPI | 0.03 ± 0.04 | ns | ns | 0.026 ± 0.04 | ns | ns | 0.007 ± 0.039 | ns | ns |
| VCAM-1 | 0.069 ± 0.015 | *** | *** | 0.067 ± 0.015 | *** | *** | 0.061 ± 0.015 | *** | ** |
|  |  |  |  |  |  |  |  |  |  |

*** = P < 0.001, ** = P < 0.01, * = P < 0.05, ns = P > 0.05. The same nomenclature is used for significance after multiple testing correction (q values)

| **Supplementary Table 8. Brief overview of the biology of the urine protein biomarkers uncovered in this lupus nephritis study** | | |  |
| --- | --- | --- | --- |
| **Marker** | **Pathways** (from KEGG and REACTOME) | **Function** (from UniProt) | **Location (**from REACTOME) |
| **ALCAM** | Axion Guidance; Cell Adhesion | Cell adhesion molecule that mediates both heterotypic cell-cell contacts via its interaction with CD6, as well as homotypic cell-cell contacts; Promotes T-cell activation and proliferation via its interactions with CD6; Contributes to the formation and maturation of the immunological synapse via its interactions with CD6 | Plasma membrane |
| **BFL-1** | NF-kappa B signaling; Apoptosis; Transcriptional misregulation in cancer; Acute myeloid leukemia | Retards apoptosis induced by IL-3 deprivation. May function in the response of hemopoietic cells to external signals and in maintaining endothelial survival during infection. Can inhibit apoptosis induced by serum starvation in the mammary epithelial cell line HC11 | Cytoplasm/Cytosol |
| **Calpastatin** | Neurodegenerative diseases; extracellular matrix organization | It exerts specific inhibition of calpain (calcium-dependent cysteine protease). Plays a key role in postmortem tenderization of meat and have been proposed to be involved in muscle protein degradation in living tissue. | Cytoplasm/Cytosol |
| **FcgRIIBC** | Phagosome; Osteoclast; B cell receptor signaling; Fc gamma R-mediated phagocytosis; Tuberculosis; Staph Aureus infection | Receptor for the Fc region of complexed or aggregated immunoglobulin gamma. Low affinity receptor. Involved in a variety of effector and regulatory functions such as phagocytosis of immune complexes and modulation of antibody production by B-cells. Binding to this receptor results in down-modulation of previous state of cell activation triggered via antigen receptors on B-cells (BCR), T-cells (TCR) or via the Fc receptor. | Plasma membrane |
| **Hemopexin** | Vesicle-mediated transport; Scavenging of heme from plasma | Binds heme and transports it to the liver for breakdown and iron recovery, after which free hemopexin returns to the circulation. May also play a role in inflammation. | Extracellular region |
| **MCP-1** | Cytokine-cytokine receptor interaction; chemokine signaling; NOD-like receptor signaling; IL-17 signaling; TNF signaling | Acts as a ligand for C-C chemokine receptor CCR2; Signals through binding and activation of CCR2 and induces strong chemotactic response and mobilization of intracellular calcium ions; Exhibits chemotactic activity for monocytes and basophils but not neutrophils or eosinophils. | Extracellular region |
| **Peroxiredoxin** | Peroxisome signaling; Oxidative stress | Thiol-specific peroxidase that catalyzes the reduction of hydrogen peroxide and organic hydroperoxides to water and alcohols, respectively. Plays a role in cell protection against oxidative stress by detoxifying peroxides and as sensor of hydrogen peroxide-mediated signaling events. | Cytoplasm/Cytosol |
| **PF-4** | Cytokine-cytokine receptor interaction; chemokine signaling; viral protein interaction | Released during platelet aggregation. Neutralizes the anticoagulant effect of heparin because it binds more strongly to heparin than to the chondroitin-4-sulfate chains of the carrier molecule. Chemotactic for neutrophils and monocytes. Inhibits endothelial cell proliferation. | Extracellular region |
| **Properdin** | Alternate complement pathway; role in HSV1 infection | A positive regulator of the alternate pathway of complement. It binds to and stabilizes the C3- and C5-convertase enzyme complexes. | Extracellular region |
| **sE-Selectin** | Cell Adhesion (at vascular wall); TNF signaling; AGE-RAGE signaling | Cell-surface glycoprotein having a role in immunoadhesion. Mediates the adhesion of blood neutrophils in cytokine-activated endothelium through interaction with SELPLG/PSGL1. May have a role in capillary morphogenesis. | Plasma membrane |
| **TFPI** | Complement and coagulation cascades | Inhibits factor X (X(a)) directly and, in a Xa-dependent way, inhibits VIIa/tissue factor activity, presumably by forming a quaternary Xa/LACI/VIIa/TF complex. It possesses anti-thrombotic action and also the ability to associate with lipoproteins in plasma. | Extracellular region |
| **VCAM1** | NF-kappa B signaling; Cell Adhesion; TNF signaling; AGE-RAGE signaling; Leukocyte transendothelial migration | Important in cell-cell recognition. Appears to function in leukocyte-endothelial cell adhesion. Interacts with integrin alpha-4/beta-1 (ITGA4/ITGB1) on leukocytes, and mediates both adhesion and signal transduction. VCAM1/ITGA4/ITGB1 interaction may play a pathophysiologic role both in immune responses and in leukocyte emigration to sites of inflammation. | Plasma membrane |

**C**

**B**

**A**

**Supplementary Figure 1.** 1129 proteins were interrogated in urine samples using an aptamer-based proteomic screen, in 24 subjects, and normalized by urine creatinine. The proteins that were significantly elevated in the urine of patients with active LN clustered into 20 pathways with at least 10 upregulated proteins each, as determined by Ingenuity Pathway Analysis, of which 3 are displayed. Molecules elevated in LN urine are shaded pink. Documented and putative interactions between the displayed molecules are indicated by solid and interrupted arrows, respectively, based on literature review.


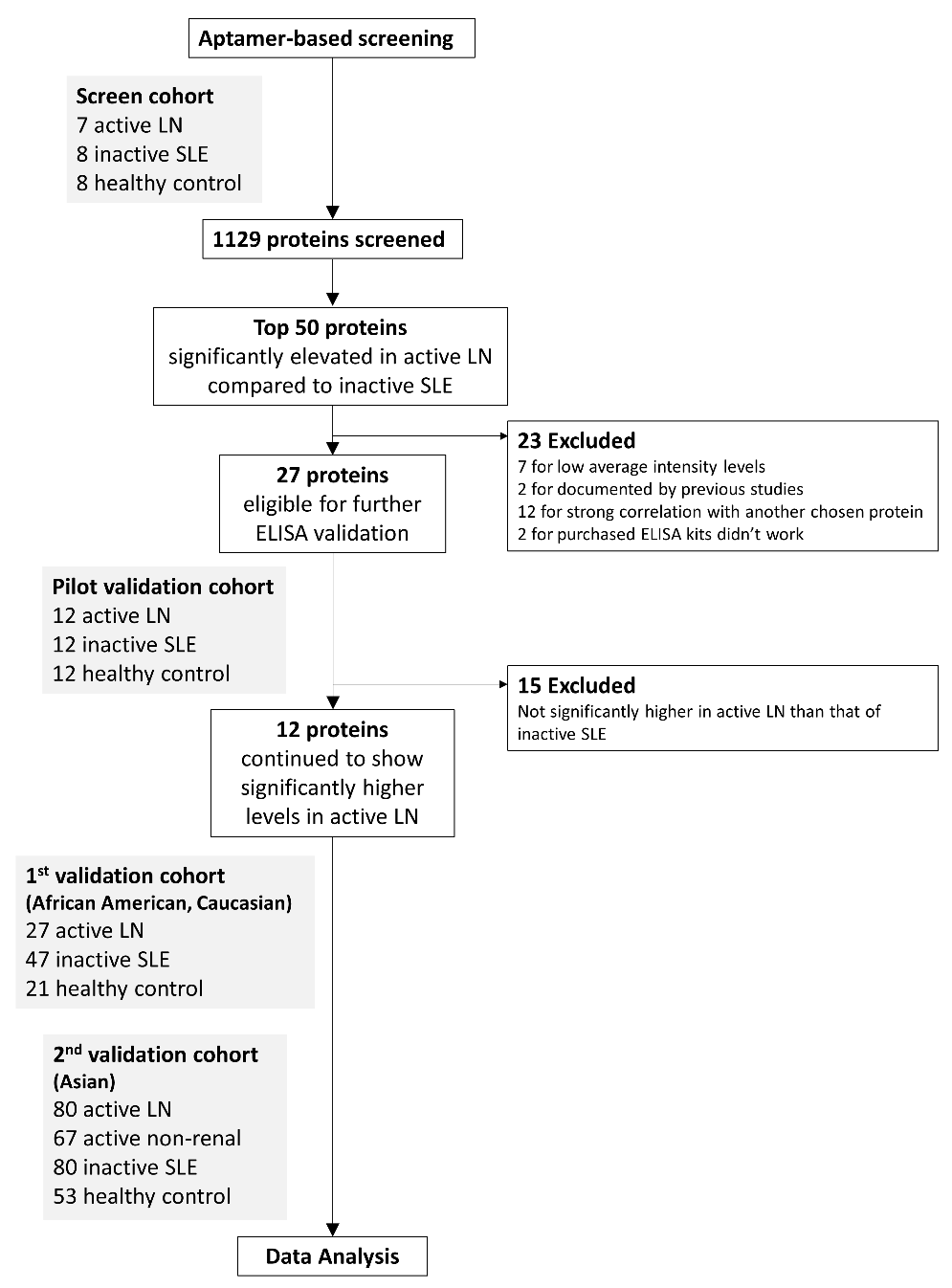


**Supplementary Figure 2.** The flowchart of study.


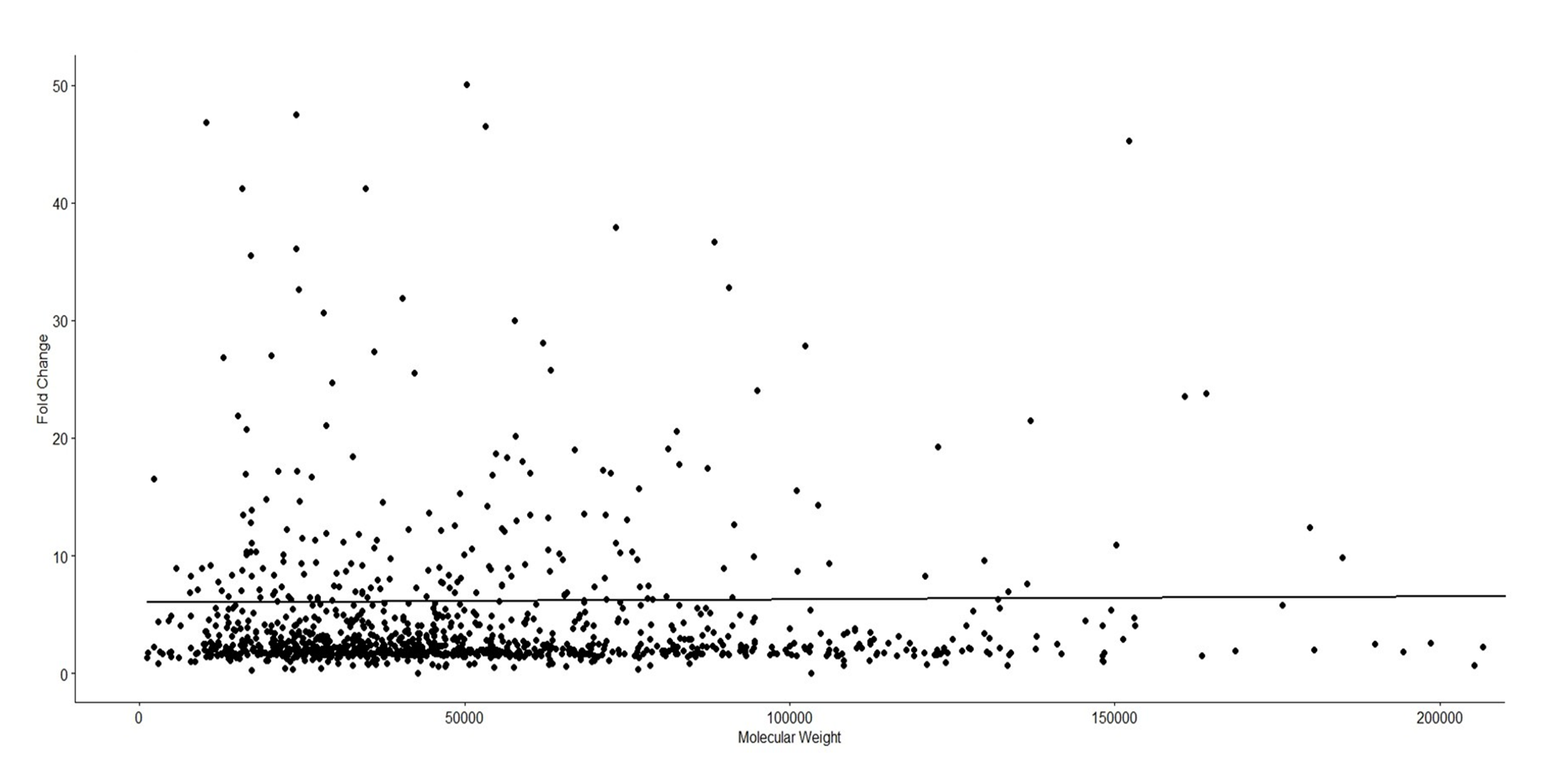


**Supplementary Figure 3.** 1129 proteins were interrogated in urine samples using an aptamer-based proteomic screen, in 24 subjects, and normalized by urine creatinine. Plotted are the fold-change (active LN versus controls) versus the molecular weights of the proteins, and the corresponding regression line. Not shown are 42 outlier proteins that exhibited fold-change > 50 or molecular weight > 200,000.


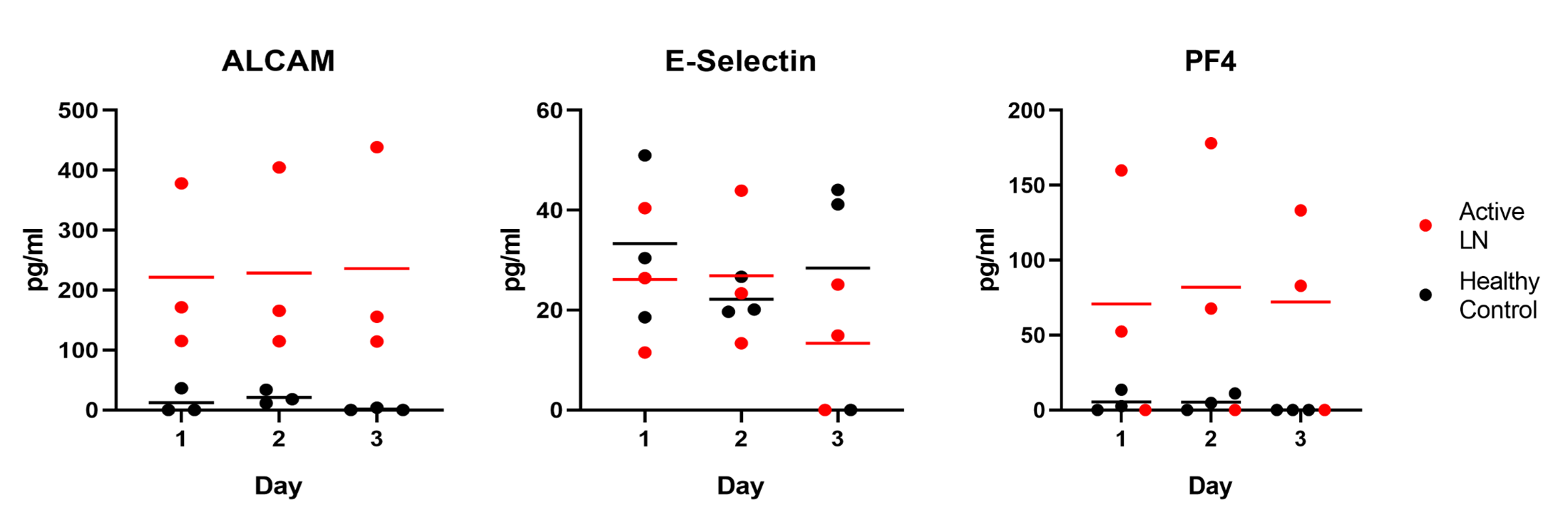


**Supplementary Figure 4**. Three proteins, ALCAM, E-selectin, PF-4, were measured in three consecutive days in three active LN patients and three healthy controls. Each dot represents the urine concentration of the protein of an individual subject.
