# Supplementary material for: Comprehensive aptamer-based screening identifies a spectrum of urinary biomarkers of lupus nephritis across ethnicities
Source: Nat Commun. 2020 May 4;11:2197. doi: 10.1038/s41467-020-15986-3 (PMC7198599; doi:10.1038/s41467-020-15986-3)
Supplement: Supplementary file 2 — Reporting Summary [file 41467_2020_15986_MOESM2_ESM.pdf]

nature research

Corresponding author(s): DBPR NCOMMS-19-15495B

Last updated by author(s): Feb 18, 2020

Reporting Summary

Nature Research wishes to improve the reproducibility of the work that we publish. This form provides structure for consistency and transparency in reporting. For further information on Nature Research policies, see Authors & Referees and the Editorial Policy Checklist.

Statistics

For all statistical analyses, confirm that the following items are present in the figure legend, table legend, main text, or Methods section.

n/a	Confirmed	
☐	☒	The exact sample size (n) for each experimental group/condition, given as a discrete number and unit of measurement
☐	☒	A statement on whether measurements were taken from distinct samples or whether the same sample was measured repeatedly
☐	☒	The statistical test(s) used AND whether they are one- or two-sided<br>*Only common tests should be described solely by name; describe more complex techniques in the Methods section.*
☐	☒	A description of all covariates tested
☐	☒	A description of any assumptions or corrections, such as tests of normality and adjustment for multiple comparisons
☐	☒	A full description of the statistical parameters including central tendency (e.g. means) or other basic estimates (e.g. regression coefficient) AND variation (e.g. standard deviation) or associated estimates of uncertainty (e.g. confidence intervals)
☐	☒	For null hypothesis testing, the test statistic (e.g. F, t, r) with confidence intervals, effect sizes, degrees of freedom and P value noted<br>*Give P values as exact values whenever suitable.*
☒	☐	For Bayesian analysis, information on the choice of priors and Markov chain Monte Carlo settings
☒	☐	For hierarchical and complex designs, identification of the appropriate level for tests and full reporting of outcomes
☒	☐	Estimates of effect sizes (e.g. Cohen's d, Pearson's r), indicating how they were calculated

Our web collection on statistics for biologists contains articles on many of the points above.

Software and code

Policy information about availability of computer code

Data collection	No software was used to collect the data.
Data analysis	Biomarker data was plotted and analyzed using either GraphPad Prism 5 (GraphPad, San Diego, CA), Microsoft Excel 2016 (Microsoft Corporation), or MATLAB R2016 (Natick, MA), or R 3.4.1 (R Foundation for Statistical Computing, Vienna, Austria).

For manuscripts utilizing custom algorithms or software that are central to the research but not yet described in published literature, software must be made available to editors/reviewers. We strongly encourage code deposition in a community repository (e.g. GitHub). See the Nature Research guidelines for submitting code & software for further information.

Data

Policy information about availability of data

All manuscripts must include a data availability statement. This statement should provide the following information, where applicable:

- Accession codes, unique identifiers, or web links for publicly available datasets
- A list of figures that have associated raw data
- A description of any restrictions on data availability

The source data is freely available by contacting the communicating author, or as source files.: http://hoc.bme.uh.edu/services/targeted-proteomics/examples-of-proteomic-screens/

Field-specific reporting

Please select the one below that is the best fit for your research. If you are not sure, read the appropriate sections before making your selection.

☒ Life sciences ☐ Behavioural & social sciences ☐ Ecological, evolutionary & environmental sciences

For a reference copy of the document with all sections, see nature.com/documents/nr-reporting-summary-flat.pdf

Life sciences study design

All studies must disclose on these points even when the disclosure is negative.

Sample size	power calculations were used to predetermine the sample size
Data exclusions	No data were excluded from analyses.
Replication	All attempts of replication were successful.
Randomization	Samples were allocated into each group according to the clinical disease status.
Blinding	Biomarker assays were done by investigators who were blinded to the diagnosis or subject group.

Reporting for specific materials, systems and methods

We require information from authors about some types of materials, experimental systems and methods used in many studies. Here, indicate whether each material, system or method listed is relevant to your study. If you are not sure if a list item applies to your research, read the appropriate section before selecting a response.

Materials & experimental systems

n/a	Involved in the study	
☒	☐	Antibodies
☒	☐	Eukaryotic cell lines
☒	☐	Palaeontology
☒	☐	Animals and other organisms
☐	☒	Human research participants
☒	☐	Clinical data

Methods

n/a	Involved in the study	
☒	☐	ChIP-seq
☒	☐	Flow cytometry
☒	☐	MRI-based neuroimaging

Human research participants

Policy information about studies involving human research participants

Population characteristics	We included two independent cohorts with three different ethnicities. Subject characteristics are described in table 2 and supplementary table 5.
Recruitment	Patients were recruited from the University of Texas Southwestern Medical Center's Renal Clinic, Dallas, TX, Johns Hopkins University School of Medicine, Baltimore, MD or Tuen Mun Hospital, Hong Kong, China, during their regular visits to these medical centers. All patients gave informed consent.
Ethics oversight	Johns Hopkins University School of Medicine, UT Southwestern Medical Center, Tuen Mun Hospital, and the University of Houston.

Note that full information on the approval of the study protocol must also be provided in the manuscript.
